# Heritage language maintenance in the Inner Circle: a scoping review of Chinese and its varieties

**DOI:** 10.3389/fpsyg.2025.1719014

**Published:** 2026-02-23

**Authors:** Xinrui Song

**Affiliations:** School of Education, College of Arts, Law and Education (CALE), University of Tasmania, Launceston, TAS, Australia

**Keywords:** Chinese languages, heritage language maintenance, Inner Circle, research synthesis, scoping review

## Abstract

Studies on heritage language have emerged from second language acquisition studies in recent decades. Researchers have become increasingly interested in attitudes, practices and challenges toward heritage language maintenance. This study examines this domain with a focus on Chinese and its varieties in Inner Circle countries, which include United States, Canada, United Kingdom, Ireland, Australia and New Zealand. This study follows research synthesis framework of a scoping review, systematically searching and examining research contexts, focused population, research trend, and the use of the term “Chinese” in existing empirical research in heritage language maintenance domain. Through a systematic search of six databases, 28 empirical studies (all journal articles) were included in this review for meeting eligibility criteria. Two reviewers along with the author individually screened and coded this sample using the coding scheme modified for this review, then cross-checked coding. Frequencies and percentages of quantitative data were then calculated, followed by the examination of qualitative data. Among other results, this study revealed a keen research interest in home, community and/or heritage language school context compared to formal governmental school settings across Inner Circle countries. The imprecise use of the term “Chinese” in the sample of this study also indicated the evident “Chinese equals Mandarin” discourse and conflation of people’s multifaceted cultural and linguistic identities. This review attempts to depict this growing domain by highlighting research trends in Inner Circle countries, identifying research gaps, and pointing potentially fruitful directions for future research.

## Introduction

Since the end of last century, language dominance and language shift have been attracting researchers’ interests worldwide (e.g., Fishman, 1991a, 1991b; Phillipson, 1992). Previous research defines language dominance as a phenomenon that one language dominating the others during interaction due to the larger population of speakers, higher status and prestige attached to the language, typically occurs between two historically distinguished groups of language users (Bishop, 2007; Phillipson, 1992). Consequently, the dominant group prioritizes their language over the others as the incentive to adopt the dominated language is no longer present (Bishop, 2007; Spolsky, 2004). Meanwhile, the prestigious group leverages the dominant language to further exploit marginalized groups with fewer resources and lower status, continuously pressuring them to adopt the dominant language and its underlying ideologies (Phillipson, 1992; Phillipson and Skutnabb-Kangas, 2012, 2017). This creates a self-reinforcing cycle that reproduces and solidifies the unequal division of resources and power. Researchers have recognized the significant and dominant role English plays in international communications, relations, academic advancement, and global business has made English a modern *lingua franca*, which is a common language requirement for pursuing higher education and employment opportunities (Hildebrandt-Wypych and Czech-Włodarczyk, 2018; Galloway and Rose, 2015; Kachru, 1985; McArthur, 2002) Kachru, (1985)‘s three circles model allocated United States, United Kingdom, Ireland, Canada, Australia and New Zealand in the Inner Circle of a concentric circle model, for the dominance of English language and culture in these countries; distinguished from the outer circle, in which those countries were colonized by the British and once dominated by English language and culture, such as India and Nigeria; and the expanding circle for countries historically made limited contact with English culture and language, such as China and Russia. Despite the dominance of English language and culture in the Inner Circle countries, these countries are not assumed to be monolingual but close to the extreme of monolingualism on the spectrum of multilingualism compares with the Outer and Expanding Circles (Kachru, 2005). Fishman (2012) advocated that multilingualism is an urgent and valid goal for multifaceted and multi-cultural citizenship in today’s globalised world, as languages and cultures of daily intimate identity contribute to maintaining multifaceted and complexes personal and social identities. Previous research has argued that promoting multilingualism requires status planning or socio-functioning planning, which is about ‘uses of language’; acquisition planning, which is about ‘users of language’, and corpus planning, which is ‘about language’ that focuses on strengthening language defense proactively (Hornberger, 2006; Spolsky, 2012b; Fishman, 2012). Fishman (2012) categorised language defence into permissive language defence, the most elementary and modest approach to seeking a permissive stance recognized from majority authorities; active language defence, which functions as a therapeutic approach when identifying the danger of any disadvantaged language; and the last category, proactive defence, which is claimed to be the most effective approach that highlights any problems before getting to the language-threatening stage. Moreover, Fishman (2012) stressed that promoting proactive language schools alone is insufficient to maintain languages across generations, he advocated that family, home, neighbourhood and community must all contribute.

### Current studies on heritage language and heritage language maintenance

The term “heritage language” was first coined in Canada and then adopted in the United States in the 1990s, referring to a minority language spoken in a society where most population do not speak the language (Cummins, 2005). Alternative terms emerged from studies across the world include, community, ancestral, indigenous, immigrant, non-official and home languages (Lo Bianco, 2010; Fishman, 2006; Wiley, 2001). Extensive research indicated that adopting the same terminology across different socio-political contexts does not necessarily denote a similar target population. For example, the term “community languages” carries distinct meanings depending on context. In a European context, it refers to official national languages recognized by each member state; only when EU enacted legislation in 1992, were indigenous and non-indigenous languages spoken by ethnic minorities included in extended recognition of the initiatives (Coulmas, 1991; Modiano, 2022). In contrast, Australia includes both indigenous and immigrant languages under the umbrella term “community languages,” aligning with the definition of heritage language as any language other than English spoken by community members in the North American context (Cruickshank et al., 2020; Montrul, 2015, 2023). As the definition of heritage language is predominantly based on local sociolinguistic context, studies have been investigating the complex linguistic and cultural identities of speakers and users in a particular context, making it challenging to offer a definition that fits all heritage language speakers, learners and users’ distinguished cultural and linguistic backgrounds (see Polinsky and Kagan, 2007; Valdés, 2005; Duff and Doherty, 2019). Researchers have proposed two conceptions of heritage language, narrow and broad, based on Fishman’s (2001, p.81) emphasis of family relevance of the language and (Van Deusen-Scholl, 2003) definition of heritage language learners, who “have been raised with strong cultural connection and heritage motivation to a particular language through family interactions.” Despite significant momentum offered by family relevance and heritage motivation, (Valdés, 2001) highlighted distinct characteristics that distinguish heritage language speakers from learners. Her most frequently quoted definition of heritage speaker composites three crucial characteristics (Valdés, 2000) (see Appendix A), taking bilingual competency into consideration and highlighting the significance of proficiency level in defining a heritage speaker.

Extensive research has investigated heritage language maintenance from a cultural and socio-psychological perspectives. Studies have explored the competition between home and ethnic ideologies and dominant ideology (Heath, 1983; Tollefson, 1991; Fillmore, 2000; Piller and Gerber, 2021; Montrul, 2023), while others have investigated language learners constantly negotiate the use of their heritage language alongside the dominant language in everyday forms of power relations (Hornberger and Wang, 2008; Zhou and Liu, 2023).

### The case of Chinese and its varieties as a HL in the inner circle context

Chinese immigrants have become one of the fastest-growing communities in Inner Circle countries, originating from the Expanding Circle [Australian Bureau of Statistics (ABS), 2016; Stats NZ, 2018; Statistics Canada, 2022; United Census Bureau, 2015; Office for National Statistics, 2021; Scotland’s Census, 2021]. These immigrants and their children face a significant struggle shaped by conflicting ideologies: government and educational sectors promote the expectation that immigrants “must” transition from their heritage languages to English; while immigrant communities maintain a strong desire to preserve their language and culture. This tension places Chinese immigrants at a crossroads between complete assimilation into English language and culture, which is the dominant societal expectation, and making deliberate efforts to maintain their heritage language, such as incorporating family language policy [FLP] at home. Curdt-Christiansen (2006, 2009, 2018) developed a dynamic Family language policy model to illustrate the interplay of FLP and its complex socio-cultural-political-linguistic environment (e.g., one parent, one language strategy; only heritage language at home strategy). This dilemma occurs during migration from the outer and expanding circle towards the Inner Circle, where English serves as the dominant language in daily, educational, and governmental contexts (Phillipson and Skutnabb-Kangas, 2012, 2017).

As one of the most rapidly growing migrant communities moving from the Expanding Circle towards the Inner Circle, Chinese immigrants have attracted researchers’ increasing attention in investigating the current state of learning and maintaining its heritage languages in specific countries and regions. However, the varieties of Chinese languages spoken within the community have been shifting due to the changing and increasingly diverse demographics of Chinese immigration, which now draws from a broader geographic range of China. Simultaneously, the promotion of Mandarin as “standard” Chinese both in China and globally fosters a unified sense of belonging to an officialised language while continuing to marginalize other Chinese varieties, granting Mandarin privileged status in educational and governmental sectors.

Research interest in Chinese heritage languages [CHLs] has evolved from second language acquisition studies, such as (Lee, 2002) survey examining the significance of cultural and linguistic identity on Chinese and Korean American students’ academic achievement, to investigations of classroom discourses in Saturday Chinese-learning school (Curdt-Christiansen, 2006), and subsequently to studies defining and exploring family language policy of Chinese immigrant families in North America (Curdt-Christiansen, 2009; King et al., 2008; Spolsky, 2012a). While most of this early research focused on Mandarin, some of the studies refer to the language as “Chinese” without specifying the particular variety of CHL. Extensive studies have investigated the language shift among Chinese language varieties in the North America: during 19th century to 1950s, “Chinese education” in North America referred to Hoisan-wa (mostly); then shifted to Cantonese in the latter half of 20th century due to the influx immigration wave from Hong Kong prior to the hand-over in 1997; only until 1990s, a considerate number of language learning schools decided to switch to Mandarin-only programs (Duff and Doherty, 2019; Duff and Li, 2013; Leung, 2011). Similar language constitution shift was also evident in the Australian context, as researchers highlighted that the original Chinese migrants (1840s–1970s) spoke Cantonese and established the first community language school in the 1880s for children of Cantonese-speaking market gardeners and restaurant workers in Sydney; whereas, more recent immigrants are constituted with Mandarin speakers from broader areas of China (Cruickshank et al., 2020; Lo Bianco, 2009). However, similar studies investigating the relationship between language shift among CHLs and language policy and planning in other Inner Circle countries are comparatively limited. Additionally, while studies have emphasized the significant role of family investment, capital and relations in maintaining CHLs in Australian contexts (Mu, 2014, 2015; Mu and Dooley, 2015; Tannenbaum and Howie, 2002; Cruickshank et al., 2020), further research is needed to examine current roles of families, community and/or weekend language schools and formal schools in heritage language maintenance across Inner Circle countries for promoting a comprehensive understanding while contributing to support and promote reviewing and modification of current heritage language policies and practices.

### Previous reviews and current study

This scoping review runs parallel with Visonà and Plonsky’s (2020) scoping review on Arabic as a heritage language, as their review provided a systematic review design for researchers to utilise for different research interests within the heritage language synthesis domain. To date, Jiang and Troyan (2022) provides the only research synthesis on a variety of CHLs, reviewing 25 empirical studies worldwide with a perspective to investigate issues of marginalized varieties of Chinese while exploring definitions of heritage language learning in existing literature and current research trend in both formal school programs, informal community and family context. This review therefore builds and expands on Jiang and Troyan’s (2022) review by focusing on maintaining Chinese and its varieties through a systematic examination of research contexts, focused population, main research interests, focused Chinese language varieties and adopted terminologies. Unlike Visonà and Plonsky's (2020) scoping review, which focused on a specific heritage language, De Souza et al. (2022) conducted a scoping review to examine how immigrant parents and children navigate a context with two languages. Both reviews employed a parallel review design that exemplifies the characteristics of a scoping review within the research synthesis community. Scoping reviews, such as the current review, offers a systematic overview of existing literature by examining volume, characteristics and trends of primary research. Using transparent, rigorous and reproducible methods, these reviews comprehensively identify and analyse available empirical to identify key concepts underpinning a specific research area (Arksey and O’Malley, 2005; Munn et al., 2018; Pham et al., 2014). The current scoping review adopts a similar review design in exploring the status of literature of maintaining Chinese and its varieties as a heritage language in the Inner Circle.

Due to the evolving demographics and linguistic diversity within the Chinese immigrant community and the broad research landscape of the Inner Circle, the exploratory nature of scoping review aligns seamlessly with the research objectives of this study. This study employs a broad conception of heritage language, one that encompasses both heritage motivation and family relevance to a language (Polinsky and Kagan, 2007) to include existing research that defines heritage language based on family relevance and language motivation emerged from possible connection to cultural and linguistic heritage. This study aims to systematically map out existing literature on maintaining Chinese and its varieties as a heritage language across the Inner Circle countries since 2000 onward; and attempt to identify research trends and gaps across the Inner Circle in a systematic manner; and highlight insufficient attention to varieties beyond Mandarin. Through depicting and comparing the body of heritage language maintenance research on Mandarin and other varieties of Chinese, I hope to fill gaps in cross-national comparative research, promote understanding of linguistic diversity within Chinese communities, and identify research gaps for further studies on other varieties of Chinese that have been overlooked or sacrificed due to heavy emphasis on Mandarin maintenance.

In this scoping review, the author addresses the following research questions:

1. What are the research contexts and populations of focus in existing literature?
2. What are the author-provided keywords and main research interests in existing literature?
3. Which varieties of Chinese heritage languages are investigated in existing literature, and which terminologies are used?

## Methods

### Studies identification and selection

This review employed a mixture of quantitative and qualitative research methods to systematically investigate empirical studies of CHLs in the Inner Circle countries. This review was conducted in accordance with the Joanna Briggs Institute [JBI]‘s reviewers’ manual, which was developed based on Arksey and O’Malley (2005) five-stage methodological framework and further enhancements proposed by Levac et al. (2010). This study’s methodological approach is based on Arksey and O’Malley (2005) five-stage methodological framework, which was originally proposed for social sciences research. The methodology is further enhanced by integrating JBI’s population/concept/context framework, which has been a widely utilized approach to enhance the systematic identification and organisation of review parameters (Peters et al., 2020; e.g. Ramírez et al., 2021; De Souza et al., 2022).

This review was broadly interested in empirical studies of CHLs in the Inner Circle context, and therefore adopted a set of unrestricted search terms for title, abstract and key word searches: 1. (Mandarin OR Chinese) AND maint* AND “heritage language”; 2. (Mandarin OR Chinese) AND maint* AND “home language”; 3. (Mandarin OR Chinese) AND maint* AND heritage (speaker* OR learner* OR language learner*). Only English-language publications were eligible for this study, this review aims to map the breadth of existing literature efficiently, including non-English publications would have required translation resources, which is beyond the scope of this study. Meanwhile, the primary focus of this review is the Inner Circle, where academic research is predominately published and disseminated in English, making English-language sources the most representative of the focused research context. The search time window of this study is from 2000, when the research interest in heritage language started to emerge. Other eligibility criteria incorporated in this review include: peer-reviewed, empirical journal articles (both published and in press) and doctoral dissertations; geographical locations of research included in Kachru’s (2005) Inner Circle framework (i.e., UK, US, Ireland, Canada, Australia and New Zealand); a focus on maintaining at least one of the CHLs, such as Mandarin and Cantonese, or studies that focus on heritage language including CHLs and other heritage languages. After consultation with the librarian, the author applied these search terms for an initial search of literature collected in UTAS Mega Search, a search engine produced by EBSCO to identify studies from major databases that UTAS subscribes and grants access to. In the initial search, five databases were identified. After further consultation with the librarian, the author applied the set of search terms in a systematic search of literature collected in six databases: Education Source, Academic Search Ultimate, Communication Source, Educational Resources Information Center (ERIC), Scopus and Web of Science. The author selected these databases as they collect scholarly literature, dissertations/theses, conference proceedings in the social sciences and have been widely incorporated in previous syntheses studies in heritage language research (see Ramírez et al., 2021; Visonà and Plonsky, 2020). An updated search utilizing ‘snowball’ searching technique was also adopted to screen citations included in studies if they were relevant to this review, this technique has been widely used in reviews for identifying potentially relevant studies from eligible studies and previous reviews (see Pham et al., 2014). The final search date of the databases is 2nd October, 2023.

**Figure 1:**
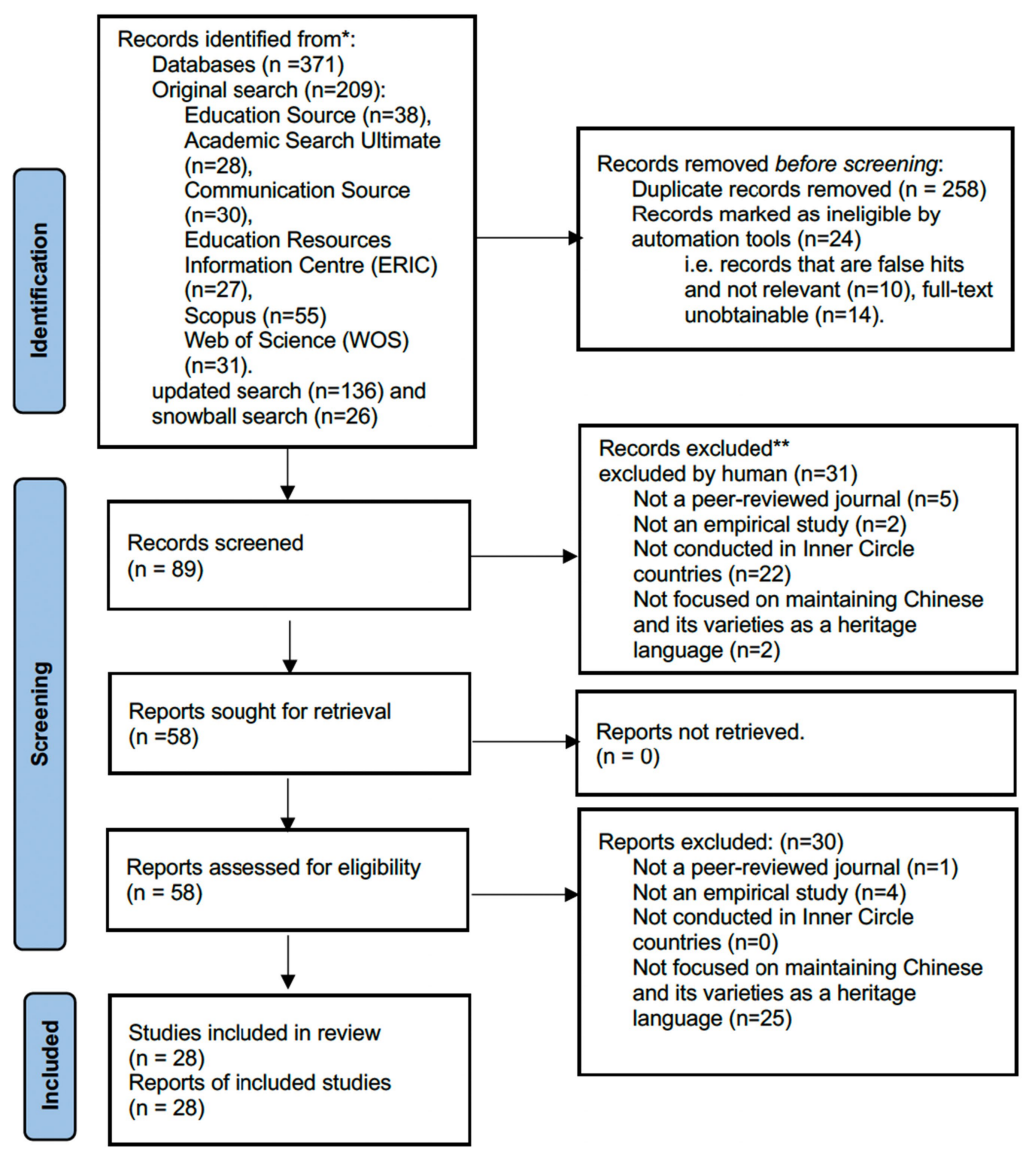
PRISMA flow chart (Source: Tricco et al., 2018).

The search terms yielded over three hundred hits, all identified results were exported and uploaded into Zotero 6.0.26 (reference manager, Cooperation for Digital Scholarship, 2023). After removing the duplicates, false hits and full-text unobtainable hits (e.g., studies that focused on phonology rather than maintenance of the language generally, a discussion of language policies rather than an empirical study), a total of 89 results entered the first round of screening (see Figure 1). The author then started the first round of screening with two reviewers by examining the titles and abstracts against the eligibility criteria. All result hits were chronically organized in an Excel spreadsheet and divided into two halves and shared with two reviewers individually via OneDrive. Disagreements between the author and reviewer’s screening results were highlighted and cross-checked by the third reviewer, then resolved by discussing to achieve a collective agreement. In this process, a total of 31 hits were excluded. The main excluding reason was that the research was not conducted in any Inner Circle countries or not published in a peer-reviewed journal (see Figure 1). The only doctoral dissertation was also excluded as it focused on learning on screen’s impact on dual language learners’ [DDL] L1 and L2 vocabulary development, which is more focused on multimedia learning’s mechanism in support vocabulary development of DDL.

Prior to the second round of screening, the author implemented a pilot full-text screening test to ensure comprehensive data extraction and systematic coding for addressing the research questions of this review. This approach was informed by Visonà and Plonsky (2020) data collection strategy, which emphasizes the significance of ensuring data trustworthiness. Twenty percent of all selected studies (k = 11) were randomly drawn and divided into two subsets (k1 = 5, k2 = 6) for two independent reviewers to examine the eligibility criteria. The author independently coded this sample and cross-checked with two reviewers’ coding separately, reaching an initial agreement percentage of 90.90% (10 out of 11), which was acceptable and exceeded expectation as it was evidently higher than Visonà and Plonsky's (2020) initial agreement percentage of 71% and revisited agreement percentage (81%). Following this, 58 hits were obtained and read in full text by the author to examine the inclusion criteria (*n* = 28). Studies identified to exclude were independently re-screened by the other two reviewers to ensure the trustworthiness and transparency of the screening results. A total of 28 reports were retained for the final sample of this review; the author recognizes that this sample may not represent the entity of CHLs research, as the scope of this review is limited in the Inner Circle countries. Nevertheless, the author believes that this sample at least accurately reflects the current state of research in the CHLs domain in the Inner Circle context.

### Data collection

Each study selected in the final sample was coded using the coding scheme that was pilot tested with a 20 percent subset of the sample for the second round of screening to ensure systematic coding and trustworthiness of extracted data. The adapted coding scheme was informed by Gurzynski-Weiss and Plonsky’s (2017) scoping review and updated in accordance with the JBI Manual for Evidence Synthesis (Aromataris, 2020), and modified wording after the pilot testing for clarity and accuracy. Items in the coding scheme reflect author’s interest in research design and substantive features of the sample, such as explicit definitions of “heritage language.” Given the purpose of this review is to depict the body of existing literature in the Inner Circle context, reveal the research trend in this field and navigate potential research gaps for further research, the author included items for bibliographic features (i.e., year of publication, journal of publication and country of the research undertaken), and open-value items for design and analysis features (i.e., participants, research context, key words, objectives/research questions). Table 1 displays the coding scheme, which was pilot tested for extracting data to answer research questions of this review and agreed for its effectiveness by two independent reviewers. This scheme was applied using Excel spreadsheets shared via OneDrive for data extraction of 28 studies in the final sample. To further maximize transparency and reproductivity, coding schemes and the raw data extracted for this review will be made accessible by contacting the author through email.

**Table 1:** Coding scheme adopted for this review.

Variable		Value				
Bibliographic features						
Author (s)	Open					
Year	Open					
Title	Open					
Publication Type	Journal article	Doctoral dissertation				
Design and analysis features						
Objectives	Open					
Participants (age group, gender, total number)	Open					
Country of origin	US	Canada	UK	Ireland	Australia	New Zealand
Research context	Home	Language school	Governmental school	Naturalistic	Open	
Focus on the maintenance of Mandarin as a heritage language only	Yes		No			
Focus on the maintenance of Mandarin and other varieties of Chinese	Yes (Input other varieties of Chinese)		No			
Generated findings	Open					
Substantive features						
Explicit definition of heritage language	Yes			No		
Definition of heritage language	Open					

### Analysis

The analysis for this review was relatively straightforward, I calculated frequencies and percentages for the values of variables relevant to each research questions, following previous scoping reviews and synthesis methods (e.g., De Souza et al., 2022; Jiang and Troyan, 2022; Visonà and Plonsky, 2020). In what follows, the author first presents an overview of bibliographical features of studies collected and coded in this review, then addressing quantitative results and qualitative findings of each of the research questions in order. The final sample consists of 28 journal articles from 18 journals, including prominent journals in bilingualism and multilingualism domains and journals that focus on a particular subfield within this domain (see Table 2). Twelve out of 28 studies were conducted in the United States, and eleven studies in Australia, together accounting for over 82% of the sample.

**Table 2:** Journals where studies of maintaining Chinese and its varieties as a heritage language in inner circle countries appear.

Journal	$K^{a}$
American Journal of Speech-Language Pathology	1
Bilingual Research Journal	4
Bilingual Review	1
Frontiers in Education	1
Frontiers in Psychology	2
HE KUPU, The Word	1
International Journal of Applied Linguistics	1
International Journal of Bilingual Education and Bilingualism	4
International Journal of Bilingualism	1
International Journal of Early Childhood Learning	1
International Journal of the Sociology of Language	2
International Multilingual Research Journal	1
Journal of Language, Identity and Education	1
Journal of Multilingual and Multicultural Development	2
Language and Communication	1
Language, Culture and Curriculum	2
Multilingua	1
Theory and Practice in Language Studies	1

K^a^ = the number of study reports.

This makes the United States and Australia the pioneering countries that have produced the most primary research of this field. The earliest studies included in this review date back to 2002 in Australia and 2003 in United States, indicating that these two countries pioneered research in this domain. Therefore, their early work may have guided and influenced subsequent studies, demonstrating their significant influence in promoting and shaping this field. Statistically, sixteen out of 28 studies were published between 2020 and 2023, with a notable surge evident in 2019–2020, indicating growing interest in this domain in recent years. This trend may be related to Covid-19 pandemic, during which online working and studying increased intergenerational interactions and conflicts within immigrant households, making them more observant in daily life. Such as 2021 study on Chinese heritage language schools in Germany focusing on migrants’ well-being during Covid-19 (Wang, 2021) study, which revealed a significant decrease in home language use during the closure of heritage language schools and absence of grandparents along with parents’ differentiated ability, confidence level and resources to support children’s multiliteracy development. A 2023 study (Li and Lin, 2023) also revealed that emergency remote learning during the Covid-19 pandemic impacted home literacy environment, parents’ literacy engagement patterns and school-home communication in complex ways. Consequently, this heightened visibility may have attracted researchers to investigate heritage language maintenance practices and policies in different contexts.

## Results

### RQ 1: What are the research contexts and populations of focus in existing literature?

All collected studies were coded into five categories of research context: home, naturalistic, community and/or weekend language schools, formal governmental school, and mixed context (see Appendix B a summary of all 28 studies). Thirteen of 28 studies (46.4%) were conducted under a mixed research context, among which home and community and/or weekend language schools were most frequently mixed. Eight studies were conducted under a naturalistic context, in which research subjects’ behaviour and conversations were observed and recorded in a real-world setting using qualitative research methods incorporating a sense of past-present-future (Mills et al., 2009; e.g. Wang, 2023; Morgan and Chodkiewicz, 2012; Diskin, 2020). Three studies were undertaken in community and/or weekend language schools alone (see Shen and Jiang, 2023; Yiakoumetti, 2022; Wu and Leung, 2022), four studies in the home context (see Wang and Obaidul Hamid, 2022b; Li, 2020; Curdt-Christiansen and La Morgia, 2018; Tang and Zheng, 2023) and none in formal governmental school settings. Despite that formal governmental schooling was mentioned in recorded conversations (see Zhang, 2009; Zhang and Slaughter-Defoe, 2009), this result stresses for reviewing the current role of formal schooling in Chinese heritage languages maintenance, contributing to a more wholistic understanding of current circumstances and potential partnership among key stakeholders in heritage language maintenance.

While most of the studies focused on a mixture of first, second and even third generation Chinese immigrants, two studies investigated Chinese-English interlingual and other cross-cultural families, only one study examined first generation Chinese immigrants’ family language policies through comparison with educational language policies and practices in formal schooling (see Kaveh and Sandoval, 2020). Meanwhile, eight studies that had offered detailed demographic features of participants revealed an overwhelmingly high involvement rate of female over males (e.g., Zhang, 2010; Wang and Hamid, 2022a; Wang and Obaidul Hamid, 2022b), as well as the willingness to undertake further interviews, including one study that only recruited mothers (see Morgan and Chodkiewicz, 2012), and no study only recruited fathers. For example, fourteen out of fifteen interviewees who expressed interest though first round online questionnaires and entered follow-up interviews were females (see Wang and Obaidul Hamid, 2022b). While this phenomenon could not represent the situation of most interviewed participants, researchers explained that such un-balanced gender distribution reflected the deeply rooted traditional pattern of labour division in some Chinese immigrant households, in which mothers are considered responsible for children’s language socialization (Wang and Hamid, 2022a).

### RQ 2: What are the author-provided keywords and main research interests in existing literature?

Each study was coded according to countries of research and manually added into reference manager software, Zotero. The author incorporated a bibliometric software called VOS viewer to illustrate the recurrence of author-provided keywords through interactive map and visualize connections between keywords and countries of research. VOS viewer is commonly utilized in bibliometric studies to generate maps based on bibliographic data exported by reference manager software (e.g., EndNote, Zotero etc.) and databases (e.g., PubMed, Scopus and Web of Science etc.), then visualize and explore maps with zooming and scrolling functionality, granting access to full details of generated maps (Van Eck and Waltman, 2023). Visualization maps generated in this review have been made available online via hyperlinks. Clicking on hyperlinks inserted beneath each visualization map directs VOSviewer Online, a website version of VOS viewer. Author-provided keywords represent the concepts and terminologies authors utilised to categorize their research, providing a lens for identifying research patterns, and suggesting varied research interests across the Inner Circle countries. With VOS viewer-generated visualization maps, thematic analysis of research interests offers complementary insights into the landscape of heritage language maintenance of CHLs in the Inner Circle.

**Figure 2:**
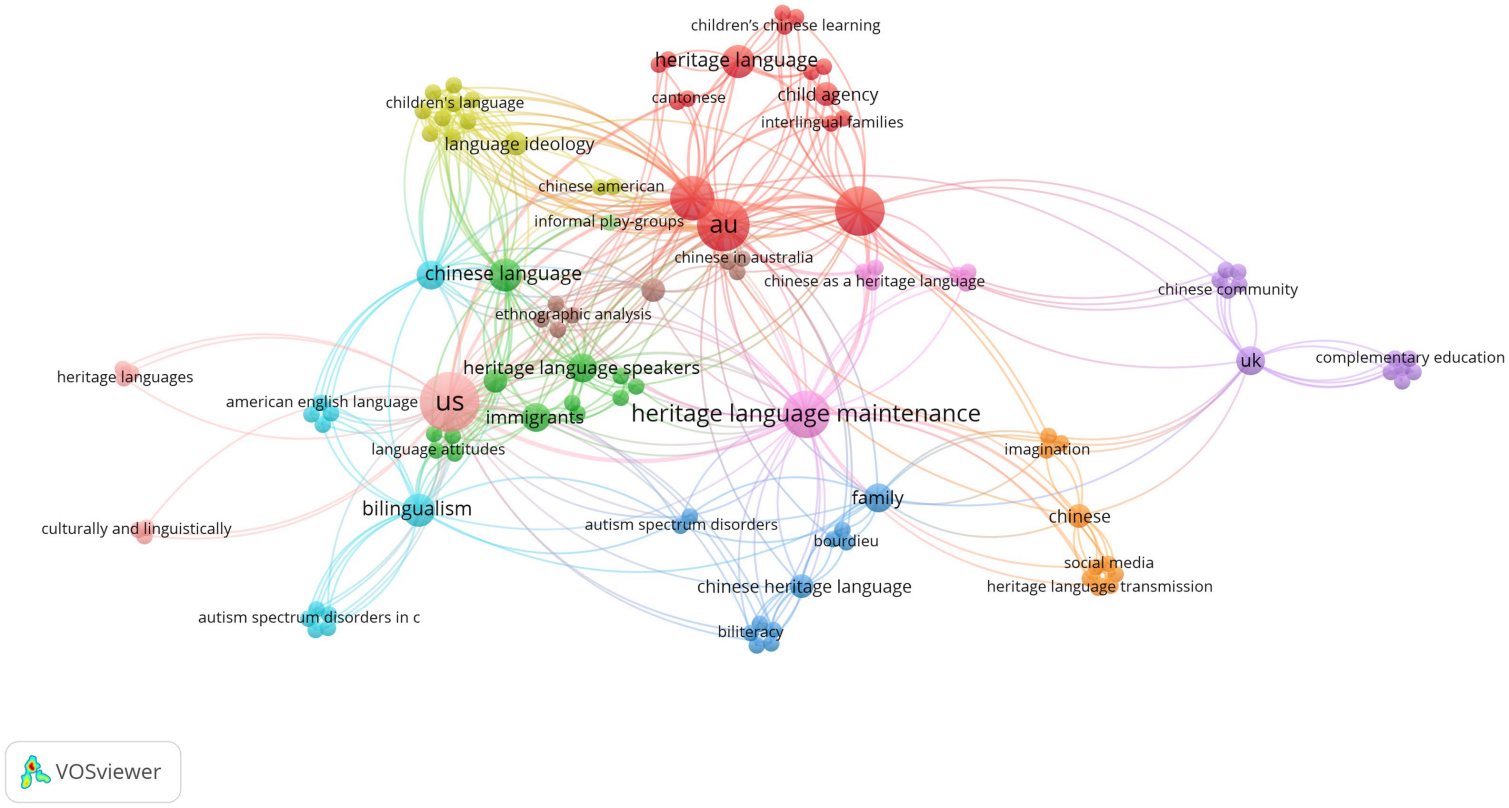
Network visualization map of reoccurrence of author-provided keywords (Source: VOSviewer online, a web-based version of VOSviewer: VOSviewer).

As illustrated in Figure 2, each color represents a cluster of author-provided key words re-occurring in the sample. Similar colors represent the membership of a cluster, following the color scale to visualize distribution of re-occurred key words. The size of the circles represents the weight and frequency of occurrence on the map. The curved lines represent the co-occurrence among key words while the link strength determines the thickness of the curved line. For example, a dense and bright red circle of “au” indicates a high value of occurrence. The curved line between “au” to “children’s language” radiates from red to green, representing progression from high to low value of occurrence.

**Figure 3:**
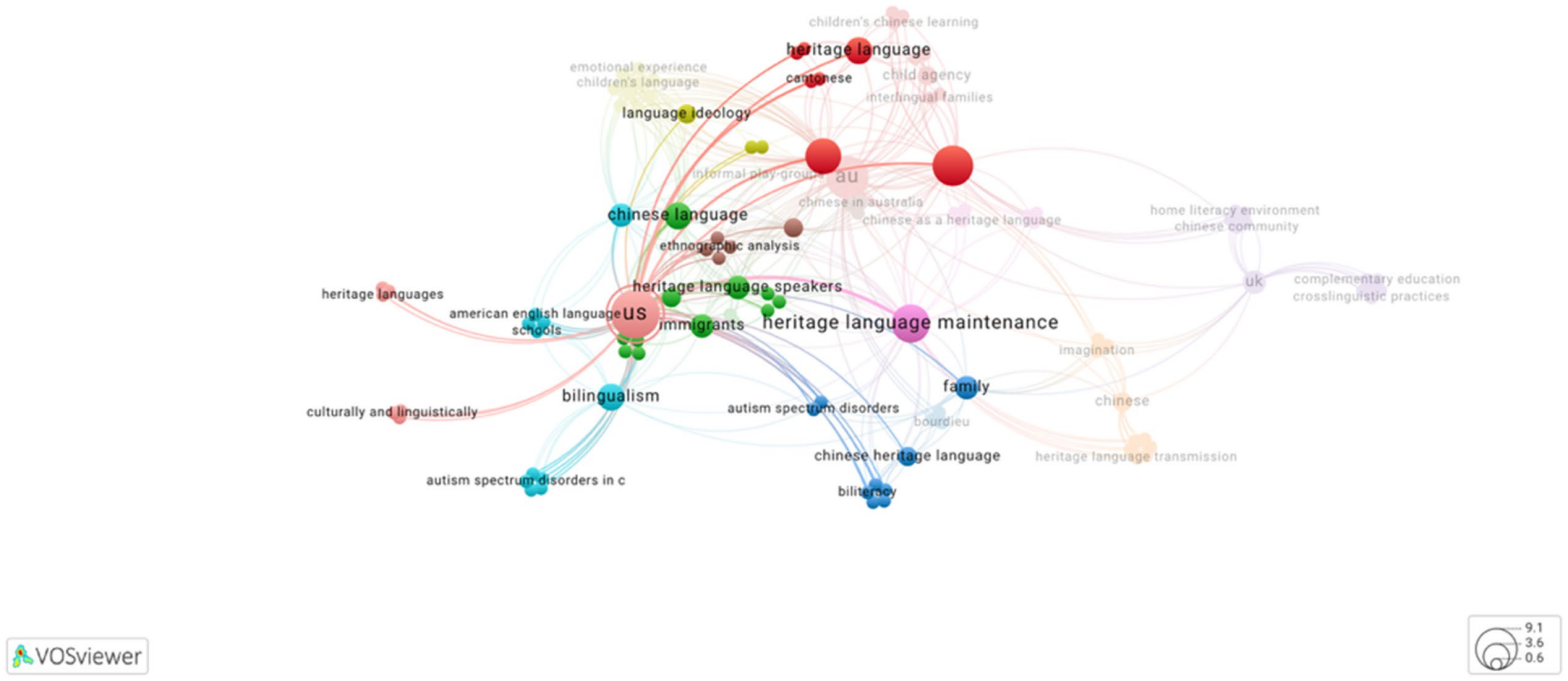
Key words that co-occurred with US (Source: VOSviewer ONLINE, a web-based version of VOSviewer: VOSviewer).

As presented in Figure 3, keywords that most frequently co-occurred with US were family language policy, heritage language maintenance, and language maintenance (overlapped in Figure 3). It is worth noting that, while the term “Chinese” occurred frequently in this sample, the key word “Cantonese” only co-occurred once with US. Upon full-texts screening, seven out of twelve studies conducted in US focused on parents’ attitudes, beliefs, thoughts, and ideological orientations towards heritage language and heritage language maintenance, comparing parents’ claimed attitudes, children’s perceptions and competence of heritage language with adopted family language policies and practices. Only two studies aimed to explore the different social networks among Mandarin, Cantonese, Hoisan-wa and Fujianese speakers, investigating the impact of these differences on negotiating and mediating cultural identities under the current “Chinese as Mandarin” discourse, as well as perceptions and practices towards heritage language maintenance.

**Figure 4:**
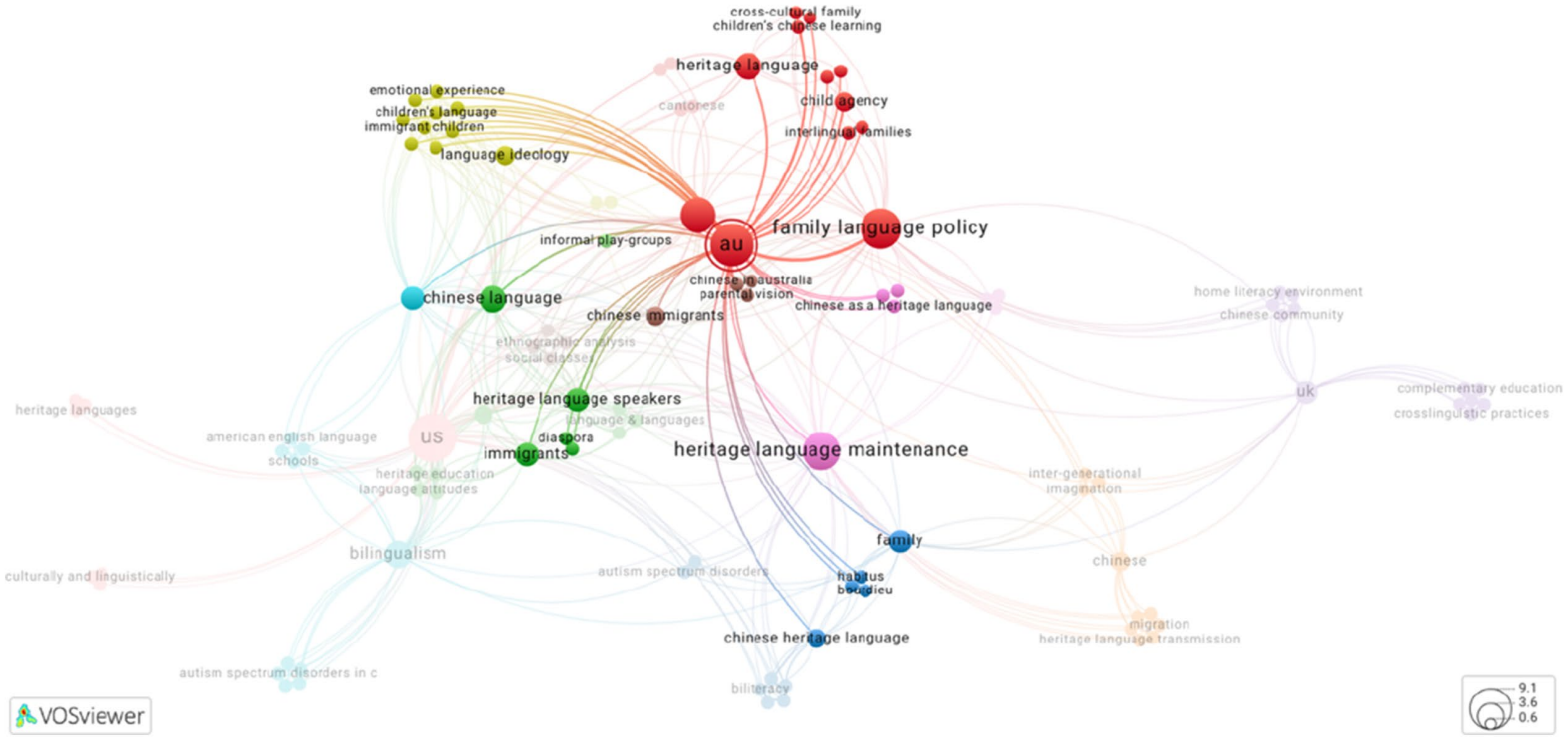
Key words that co-occurred with AU (Source: VOSviewer online, a web-based version of VOSviewer).

**Figure 5:**
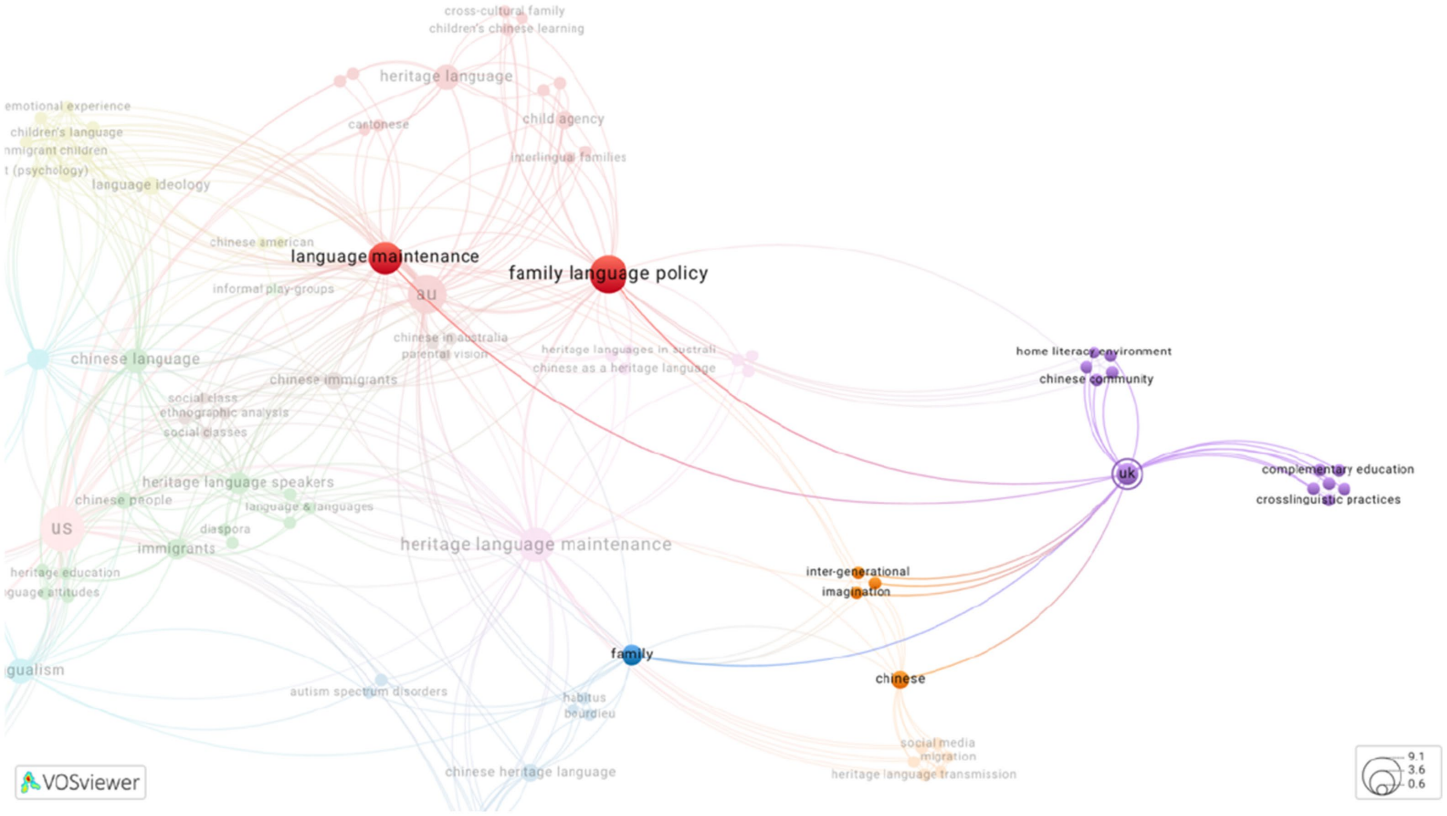
Key words that co-occurred with UK (Source: VOSviewer online, web-based version of VOSviewer).

Apart from similar research interests within the US context, studies conducted in the Australia context also contributed to research areas such as interlingual family, children’s agency and relationship between emotional experiences and language ideology (see Figure 4). Studies in the UK context shared similar interests with the Australian context, investigating community language teachers’ use of English and heritage language and their perceptions of heritage language learning as well as the influence of parental ideologies on involving in children’s language development (see Figure 5). Meanwhile, UK held the only study in this review that explored intergenerational language transmission within Cantonese and Hakka speaking families by examining the role of imagination in heritage language maintenance (Li and Zhu, 2019).

### RQ 3: Which varieties of Chinese heritage language are investigated in existing literature, and which terminologies are used?

The three most focused sub-categories of Chinese heritage languages in this sample were Chinese (k = 11), followed by Mandarin (k = 8) and Cantonese (k = 6), studies focused on Cantonese were comprised by one study that particularly focused on Cantonese and five studies that focused on Cantonese as a main component (k = 5). Studies focused on Chinese were a sum of studies only focus on Chinese (k = 4) and studies focused on Chinese and other heritage languages, including Arabic, Hebrew, Italian, Polish, Pakistani, and Spanish (k = 7). The term “Chinese” was adopted as an umbrella term without specifying the varieties of CHLs, multiple varieties of CHLs emerged from excerpt of interview records. It appeared that over-generalizing CHLs with the imprecise term “Chinese,” as well as Chinese equivalent to Mandarin discourse, remained evident in this sample (see Yu and Hsia, 2019; Zhang, 2009; Liao and Huang, 2020). Meanwhile, the remark “we Chinese” was repeatedly documented in interview records in this sample, urging a closer examination of terminologies and cultural and linguistic connotation behind this remark.

## Discussion

One of the findings of this review is that home, and community and/or weekend language schools remain the major contexts supporting CHLs maintenance across Inner Circle countries, while educational language policy of governmental school was mentioned and compared with family language policy in a UK study (see Kaveh and Sandoval, 2020), which examined influences of formal schooling on family language practices as children turn to school ages. The results suggest that maintaining CHLs in home and language school contexts has gained relatively significant and majority of empirical grounds in this domain, almost half of the 28 studies in the sample (k = 13) were based in a mixed research context, in which home, and community and/or weekend language schools were mixed most frequently, whereas other forces of society should also be reimaged in supporting and promoting this domain. As Fishman (2012) emphasized, reversing language shift and supporting intergenerational heritage language maintenance could not be achieved without having other forces of the society to contribute to higher education sphere, work sphere, and major agencies of government as well as mass media. Due to Australia’s relatively small population, geographic location and increasing economic dependence on Asian countries and non-English speaking Asian societies, Australia has come to appreciate the rapid economic growth of Asia relatively early; however, the “Asia literacy” policy and discourse was promoted based on economic regionalism instead of local presence of these languages and cultures (Liu and Lo Bianco, 2007). Consequently, political and economic factors would influence discourse planning and impact language policy and plannings through deliberate framing and naming of an issue in convincing rhetoric (Lo Bianco, 2005). Lo Bianco (2009) revealed that the intention of many bilingual programs designed and provided for Chinese immigrant families was to support the development of English proficiency and acquisition without continuous and consistent language policies and plannings (Liu and Lo Bianco, 2007). As the domain of heritage language maintenance emerging and attracting research interest, the review collected no study focused on formal schooling context, suggesting further research is urged to explore formal governmental school’s perspective and stance in CHLs maintenance.

One interesting finding is about the imprecise use of the term “Chinese” in this sample. This review found that the term “Chinese” is frequently used as an umbrella term, which includes more than one language, and each of the represented languages can be referred to as “Chinese.” Eleven studies in this sample used this term to refer to Chinese as a heritage language, while eight studies used this term to refer to Mandarin, also known as Modern Standard Chinese or Putonghua. Potential problems brought by such pluri-denominating phenomenon include conflating people, ethnicities, and languages (Leung et al., 2018), as well as solidifying the mechanism of racial regimentation by homogenizing “Chinese” immigrants regardless of their multifaceted cultural and linguistic identities (Shih, 2011). In 19th century and the first half of 20th century, people immigrated from China to US spoke Hoisan-wa and Cantonese, they referred to themselves as “Tang people” (唐人) who lived in the ghetto “Tang people’s street” (唐人街), instead of Chinese living in “China town” (中国城) (Shih, 2011). The reason why they became “Chinaman” or “Chinese” was racial assignation, the misalignment has already existed when these immigrants were assumed to be “Chinese” speakers, who in fact spoke Cantonese or other dialects instead of Mandarin. Surprisingly, in study that only focused on Cantonese, the term “Chinese” has also emerged from conversation records with interviewees who speak Cantonese and Hoisan-wa, suggesting a central orientation by making value-attributions to places they came from and places they lived in, where they only spoke Cantonese and Hoisan-wa (see Leung, 2021). Meanwhile, some interviewed third-generation immigrants, whose authentic heritage language were Cantonese or Hoisan-wa, became interested in learning Mandarin and returning to China through social networking with university friends (see Hua and Wei, 2016; Li and Zhu, 2019). This indicates the anchoring of CHLs has been shifting over generations while demonstrating new orders indexicality. Such new ordering can be caused by authenticity and understandability of the heritage language itself, promotion of Mandarin as standardized Modern Standard Chinese, and change of spatial anchoring and footing compared to first generation immigrants (Blommaert, 2005; Blommaert et al., 2005; Cruickshank et al., 2020). This finding reveals that current “Chinese as Mandarin” discourse marginalizes other CHLs outside of the centre, and is indeed arbitrary in nature, emphasizing the significance of recognizing totality of heritage speakers and heritage learners’ language repertoire, valuing all varieties apart from those that hold most prestige (see Leung, 2021; Wu and Leung, 2022). As Fishman (1991a) argued that the destruction of one’s language is indeed the destruction of one’s identity, recognizing the language repertoire allows heritage language users to fully embrace and reflect on their multifaceted cultural and linguistic identities, and to contribute to the promotion and development of language awareness.

Another interesting finding is the frequent appearance of the remark “we Chinese” in interview records with first, second and third generation immigrants in this sample. Some first-generation immigrants, who spoke Cantonese and immigrated to the UK from Hong Kong, expressed their sentiment to return to Hong Kong, and their wish for their future grandchildren to learn Chinese languages, which was referred to as “我们中国话” (Li and Zhu, 2019). Surprisingly, some third and 2.5 generation immigrants, who have never been to cities that their grandparents or parents came from, also adopted the discourse of returning to China, stating that life would be better in homeland and UK does not feel at home (see Hua and Wei, 2016; Li and Zhu, 2019). These conversations reflected some ideal, and perhaps romanticized perception of Chinese civilization, indicating that the majority of Chinese immigrants have never psychologically left China, despite the geographic distance from homeland (Redding, 2013). These sentiments indicate a sense of belonging to a unified national identity that they are all part of the “Hua Xia” ethnic group (华夏民族) or “Zhonghua” ethnic group (中华民族), which is comprised by fifty-six ethnic groups. In modern society, people who introduce themselves as “Chinese” in English, may also introduce themselves as “Hua Ren” (华人) in Chinese, meaning the people of Hua civilization. This sense of belonging to the Hua civilization and sharing the common ancestry attributes to the notion of Hua Xia-centralism, differentiating Hua Xia from foreign tribes according to shared cultural bases rather than ethnicities (Wu, 2012). Nonetheless, this sense of hierarchy of the centre was significantly weakened after the first and second Opium War in mid-19^th^ century, urging a reshaping of identity due to the profound humiliation of the defeats of two Opium Wars and semi-colonial state of the country (Wu, 2012). During the past decades, China’s rise on international stage of politics and economics allows China to step into the world’s spotlight again, reviving the wish to renaissance that once-glorious Hua Xia culture. Such renaissance also promotes Chinese immigrants’ willingness of heritage language maintenance, and it is evident that, in the sample of this review, most of participants associated more and better employment opportunities in China with the reason of maintaining and learning Mandarin, as it is the standard language commonly spoken in China. The use of the remark “we Chinese” reveals that participants’ perception of their identity is complex, evolving, and heavily relies on the constantly changing context, which is indispensable in recognizing and valuing ones’ cultural and linguistic repertoire. Therefore, the author suggests researchers to further investigate evolving cultural and linguistic identities across different generational immigrants and potential impact on family language practices of heritage language maintenance. Finally, the author urges the researcher to reflect on how language-specific synthesis contributes to the domain of heritage language maintenance. It is author’s hope that the scoping review methodology employed in this study provides a replicable framework for language-specific syntheses that effectively identify research gaps and proactively inform pedagogical improvement.

## Conclusion

This study set out to systematically examine existing literature on CHLs maintenance in the Inner Circle, with a focus on research contexts, investigated populations, author-provided keywords, research trends and variety of CHLs explored. This review revealed an increasing research interest in home and weekend and/or community language school contexts. US and Australia are the most investigated Inner Circle countries, with family language policy being the most popular topic of research. The most investigated sub-categories of CHLs are Chinese, Mandarin and Cantonese, while “Chinese” was frequently used as an umbrella term. The review also highlighted the imprecise use of the term “Chinese,” highlighting the existent “Chinese equivalent to Mandarin” discourse and the problematic conflation of people, ethnicities and languages by such pluri-denominating phenomenon. From a practical standpoint, the implications are far reaching. The results of this review indicate that further research is required to investigate intergenerational transmission across different CHLs, especially those under-investigated varieties (such as Hakka, Miao, Shanghainese etc.), to examine potential language shift occurred during intergenerational transmission while analysing factors contributing to such language shift or language maintenance. This study contributes to a deeper understanding of heritage language maintenance, though further and urgent research is required to dissect current role of formal schooling in heritage language maintenance and decode the dynamic of children/students, parents, school teachers and heritage language teachers in a triangle mechanism, of which home, formal schooling and community and/or weekend language school interact, negotiate, compete to support or hinder heritage language maintenance. The findings of this review provide an entry point for further studies that investigates the scoping status and mechanism of non-officialised varieties of heritage language maintenance in a three-party game, in which a dominant language, an officialised heritage language and a non-officialised heritage language interact, negotiate and compete. However, developing such a model requires primary empirical data, which is beyond the scope of a scoping review. In summary, the conclusion reinforces the significance of expanding research interests to prioritised research questions, populations and contexts in shaping a concrete, actionable research agenda for heritage language maintenance domain.

## References

[ref1] Arksey H. O’Malley L. (2005). Scoping studies: towards a methodological framework. Int. J. Soc. Res. Methodol. 8, 19–32. doi: 10.1080/1364557032000119616

[ref2] Aromataris E. Munn Z. (2020) in JBI manual for evidence synthesis. ed. Munn Z. (JBI). doi: 10.46658/JBIMES-20-01

[ref3] Australian Bureau of Statistics. (2016). Cultural diversity Australia: 2016 census. Available online at: https://www.abs.gov.au/articles/cultural-diversity-australia#language

[ref4] Bishop M. (2007) “An exploration of language dominance.” MA TESOL Collection 9. School for International Training. Available online at: https://digitalcollections.sit.edu/ipp_collection/9

[ref5] Blommaert J. (2005). Discourse: A critical introduction. Cambridge: Cambridge University Press. doi: 10.1017/CBO9780511610295

[ref6] Blommaert J. Collins J. Slembrouck S. (2005). Spaces of multilingualism. Lang. Commun. 25, 197–216. doi: 10.1016/j.langcom.2005.05.002

[ref7] Coulmas F. (1991). A language policy for the European Community. Berlin-New York: Mouton de Gruyter.

[ref8] Cruickshank K. Black S. Wright J. Tsung L. Chen H. (2020). Language education in the school curriculum. London: Bloomsbury Academic.

[ref9] Cummins J. (2005). A proposal for action: strategies for recognizing heritage language competence as a learning resource within the mainstream classroom. Mod. Lang. J. 89, 585–592.

[ref10] Curdt-Christiansen X. L. (2006). Teaching and learning Chinese: heritage language classroom discourse in Montreal. Lang. Cult. Curric. 19, 189–207. doi: 10.2167/lcc314.0

[ref11] Curdt-Christiansen X. L. (2009). Invisible and visible language planning: ideological factors in the family language policy of Chinese immigrant families in Quebec. Lang. Policy 8, 351–375. doi: 10.1007/s10993-009-9146-7

[ref12] Curdt-Christiansen X. L. (2018). Family language policy. The Oxford handbook of language policy and planning, eds. Tollefson J. W., and Pérez-Milans, M. New York: Oxford University Press, 420–441.

[ref13] Curdt-Christiansen X. L. La Morgia F. (2018). Managing heritage language development: opportunities and challenges for Chinese, Italian and Pakistani Urdu-speaking families in the UK. Multilingua 37, 177–200. doi: 10.1515/multi-2017-0019

[ref14] De Souza D. K. Lin H. Cox R. B. (2022). Immigrant parents and children navigating two languages: a scoping review. J. Fam. Theory Rev. 15, 133–161. doi: 10.1111/jftr.12484

[ref15] Diskin C. (2020). New speakers in the Irish context: heritage language maintenance among multilingual migrants in Dublin, Ireland. Front. Educ. 4:163. doi: 10.3389/feduc.2019.00163

[ref16] Duff P. Doherty L. (2019). “Learning ‘Chinese’ as a heritage language: challenges, issues and ways forward” in The Routledge handbook of Chinese applied linguistics, eds., Huang, C., Jing-Schmidt, Z., and Meisterernst, B. Abingdon, Oxen, New York, NY: Routledge. 149–164.

[ref17] Duff P. Li D. (2013). “Learning Chinese as a heritage language” in Minority populations in Canadian second language education, eds., Arnett, K., and Mady, C. Bristol, BS; Tonawanda, NY; North York, Ontario: Multilingual Matters. 87–100.

[ref18] Fillmore L. W. (2000). Loss of family languages: should educators be concerned? Theory Pract. 39, 203–210. doi: 10.1207/s15430421tip3904_3

[ref19] Fishman J. A. (1991a). Reversing language shift: Theoretical and empirical foundations of assistance to threatened languages, Clevedon: Multilingual matters. 76.

[ref20] Fishman J. A. (1991b). “Where’and’why’does language shift occur and how can it be reversed.”

[ref21] Fishman J. A. (2001). “From theory to practice (and vice versa): review, reconsideration an d reiteration” in Can threatened languages be saved? Reversing language shift, Revisted: A 21st century perspective, ed. Fishman, J. A. (Clevedon, England: Multilingual Matters), 451–483.

[ref22] Fishman J. A. (2006). “Three hundred-plus years of heritage language education in the United States” in Developing minority language resources: The case of Spanish in California. eds. Valdés G. Fishman J. A. Chávez R. Pérez W. (Clevedon: Multilingual Matters), 12–23.

[ref23] Fishman J. A. (2012). “Language maintenance, language shift, and reversing language shift” in The handbook of bilingualism and multilingualism. eds. Tej K. Bhatia William C. Ritchie (John Wiley & Sons).

[ref24] Galloway N. Rose H. (2015). In Reversing language shift: Theoretical and empirical foundations of assistance to threatened languages, edited by J. A. Fishman. Clevedon: Multilingual matters..

[ref25] Gurzynski-Weiss L. Plonsky L. (2017). “Look who’s interacting: a scoping review of research involving non-teacher/non-peer interlocutors” in AILA applied linguistics series. ed. Gurzynski-Weiss L.. (Amsterdam: John Benjamins Publishing Company). 16.

[ref26] Heath S. B. (1983). Ways with words: Language, life and work in communities and classrooms. Cambridge: Cambridge university Press.

[ref27] Hildebrandt-Wypych D. Czech-W C.. (2018). 7. Between linguistic diversity and the dominance of English–languages in Europe from the political, cultural and educational perspectives. In Modern foreign language learning in logistics area, eds. Adamczak, M., Domański, R., and Cyplik, P. Poznan: Publishing House of the Higher School of Logistics.

[ref28] Hornberger N. H. (2006). Frameworks and models in language policy and planning An Introduction to Language Policy: Theory and Method, ed. Ricento, M. Oxford, UK; Calton, VIC: John Wiley & Sons. 24, 41.

[ref29] Hornberger N. H. Wang S. C. (2008). Who are our heritage language learners?: Identity and Biliteracy in heritage language education in the United States In Heritage Language Education: a new field emerging, eds. Brinton, D., Kagan, O., and Bauckus, S. New York, NY, Routledge.3–35.

[ref30] Hua Z. Wei L. (2016). Transnational experience, aspiration and family language policy. J. Multiling. Multicult. Dev. 37, 655–666. doi: 10.1080/01434632.2015.1127928

[ref31] Jiang Y. Troyan F. J. (2022). Varieties of Chinese as heritage languages: a research synthesis. Int. J. Bilingual Educ. Bilingual. 27, 131–143. doi: 10.1080/13670050.2022.2159315

[ref32] Kachru B. B. (2005). Standards, codification and sociolinguistic realism: The English language in the outer circle. In English in the world: Teaching and learning the language and literatures, edited by R. Quirk and H. Widowson. Cambridge: Cambridge University Press. 3, 241–270.

[ref33] Kachru B. B. (1985). Standards, codification and sociolinguistic realism World Englishes. Critical Concepts in Linguistics, vol. 3, 241–270.

[ref34] Kaveh Y. M. Sandoval J. (2020). ‘No! I’m going to school, I need to speak English!’: The English language in the outer circle. In English in the world: Teaching and learning the language and literatures, eds. Quirk, R., and Widowson, H. Cambridge: Cambridge University Press. 43, 362–383. doi: 10.1080/15235882.2020.1825541

[ref35] King K. A. Fogle L. Logan-Terry A. (2008). Family language policy. Lang. Linguist. Compass 2, 907–922. doi: 10.1111/j.1749-818X.2008.00076.x

[ref36] Lee S. K. (2002). The significance of language and cultural education on secondary achievement: a survey of Chinese-American and Korean-American students. Bilingual Res. J. 26, 327–338. doi: 10.1080/15235882.2002.10668714

[ref9001] Leung G. Ho E. Y. Chi H.-L. Huang S. Ting I. Chan D. (2018). We (Tong) Chinese: contemporary identity positioning through health management among Cantonese Chinese Americans. J International and Intercultural Commu. 11, 271–285.

[ref37] Leung G. Y. (2011). The internet and Hoisan-Wa in the US: counter-hegemonic discourses and shifting language ideologies. J. Chin. Overseas 7, 247–257. doi: 10.1163/179325411X595422

[ref38] Leung G. (2021). Maybe useful to the future generation but not my own: how ‘useful’ is mandarin really for contemporary Hoisan-heritage Chinese Americans in the San Francisco Bay Area? Lang. Commun. 76, 121–130. doi: 10.1016/j.langcom.2020.11.003

[ref39] Levac D. Colquhoun H. O’Brien K. K. (2010). Scoping studies: advancing the methodology. Implement. Sci. 5, 1–9. doi: 10.1186/1748-5908-5-69, 20854677 PMC2954944

[ref40] Li S. (2020). ‘We only speak Chinese at home’: a case study of an immigrant Chinese family’s family language policy in New Zealand. HE KUPU 6, 41–50. Available online at: https://www.hekupu.ac.nz/article/we-only-speak-chinese-home-case-study-immigrant-familys-family-language-policy-new-zealand

[ref41] Li G. Lin Z. (2023). In and out of the unknown: lessons from immigrant families promoting multiliteracies during the COVID-19 pandemic. Read. Teach. 76, 570–577. doi: 10.1002/trtr.2184

[ref42] Li W. Zhu H. (2019). Imagination as a key factor in LMLS in transnational families. Int. J. Sociol. Lang. 2019, 73–107. doi: 10.1515/ijsl-2018-2004

[ref43] Liao W. Huang H. (2020). Parents’ perceptions and management of children’s learning of Chinese as a heritage language: a case study of cross-cultural families in Australia. Theory Pract. Lang. Stud. 10, 1218–1226. doi: 10.17507/tpls.1010.05

[ref44] Liu G.-Q. Lo Bianco J. (2007). Teaching Chinese, teaching in Chinese, and teaching the Chinese. Lang. Policy 6, 95–117. doi: 10.1007/s10993-006-9041-4

[ref9002] Lo Bianco J. (2005). Including discourse in language planning theory. In Directions in applied Linguistics, eds., Bruthiaux P. Atkinson D., Eggington W. G. Grabe W. Ramanathan V. Clevedon: Multilingual Matters, 255–264.

[ref9003] Lo Bianco J. (2009). Policy activity for heritage languages: Connections with representation and citizenship. In heritage language education: A new field emerging (1st ed.), eds., Brinton D. M. Kagan O. Bauckus S. New York: Routledge, 53–69. doi: 10.4324/9781315092997

[ref46] Lo Bianco J. (2010). “Language policy and planning” in Sociolinguistics and Language Education. eds. Hornberger N. H. McKay S. L. (Clevedon: Multilingual Matters).

[ref47] McArthur T. (2002). The Oxford guide to world English: New York: Oxford University Press.

[ref48] Mills A. J. Durepos G. Wiebe E. (2009). Encyclopedia of case study research. Thousand Oaks, California: Sage publications.

[ref49] Modiano M. (2022). EU language policy under review. Eur. J. Lang. Policy 14, 249–267. doi: 10.3828/ejlp.2022.14

[ref50] Montrul S. (2015). The Acquisition of Heritage Languages. Cambridge: Cambridge University Press.

[ref51] Montrul S. (2023). “Heritage language development: dominant language transfer and the sociopolitical context” in Studies in Italian as a Heritage Language. Scopus. ed. Romano. F. A. Berlin, Boston: Walter de Gruyter GmbH & Co KG.

[ref52] Morgan L. Chodkiewicz A. (2012). Supporting home languages in informal settings: Chinese-speaking mothers in Sydney. Int. J. Early Child. Learn. 19, 51–63. doi: 10.18848/2327-7939/cgp/v19i04/48404

[ref53] Mu G. M. (2014). Learning Chinese as a heritage language in Australia and beyond: the role of capital. Lang. Educ. 28, 477–492. doi: 10.1080/09500782.2014.908905

[ref54] Mu G. M. (2015). “Learning Chinese as a Heritage Language: An Australian Perspective” in Learning Chinese as a heritage language (Bristol, Blue Ridge Summit: Multilingual Matters). doi: 10.21832/9781783094295

[ref55] Mu G. M. Dooley K. (2015). Coming into an inheritance: family support and Chinese heritage language learning. Int. J. Bilingual Educ. Bilingual. 18, 501–515. doi: 10.1080/13670050.2014.928258

[ref56] Munn Z. Peters M. D. J. Stern C. Tufanaru C. McArthur A. Aromataris E. (2018). Systematic review or scoping review? Guidance for authors when choosing between a systematic or scoping review approach. BMC Med. Res. Methodol. 18, 1–7. doi: 10.1186/s12874-018-0611-x, 30453902 PMC6245623

[ref9004] Office for National Statistics. (2021). Main language, English language proficiency, and household language in England and Wales, Census 2021 data. Available online at: https://www.ons.gov.uk/peoplepopulationandcommunity/culturalidentity/language/bulletins/languageenglandandwales/census2021#main-languages-in-england-and-wales

[ref9005] Peters M. D. J. Godfrey C. McInerney P. Munn Z. Tricco A. C. Khalil H. (2020). Chapter 11: Scoping Reviews (2020 version). In JBI Manual for Evidence Synthesis, eds., Aromataris E. Munn Z. The Joanna Briggs Institute.

[ref57] Pham M. T. Rajić A. Greig J. D. Sargeant J. M. Papadopoulos A. McEwen S. A. (2014). A scoping review of scoping reviews: advancing the approach and enhancing the consistency. Res. Synth. Methods 5, 371–385. doi: 10.1002/jrsm.1123, 26052958 PMC4491356

[ref58] Phillipson R. (1992). Linguistic imperialism: Oxford University Press.

[ref59] Phillipson R. Skutnabb-Kangas T. (2012). “Linguistic imperialism and endangered languages” in The handbook of bilingualism and multilingualism. eds. Bhatia T. K. Ritchie W. C. (MA: Wiley-Blackwell), 495–516.

[ref60] Phillipson R. Skutnabb-Kangas T. (2017). “English, language dominance, and Ecolinguistic diversity maintenance” in The Oxford handbook of world Englishes, ed. Filppula, M., Klemola, J., and Sharma, D. New York: Oxford University Press. 312–334.

[ref61] Piller I. Gerber L. (2021). Family language policy between the bilingual advantage and the monolingual mindset. Int. J. Bilingual Educ. Bilingual. 24, 622–635. doi: 10.1080/13670050.2018.1503227

[ref62] Polinsky M. Kagan O. (2007). Heritage languages: in the ‘wild’and in the classroom. Lang. Linguist. Compass 1, 368–395. doi: 10.1111/j.1749-818X.2007.00022.x

[ref63] Ramírez R. Huang B. H. Palomin A. McCarty L. (2021). Teachers and language outcomes of young bilinguals: a scoping review. Lang. Speech Hear. Serv. Sch. 52, 755–768. doi: 10.1044/2020_LSHSS-20-00066, 33751903

[ref64] Redding G. (2013). The Spirit of Chinese capitalism, Berlin, New York: Walter de Gruyter. 22.

[ref9006] Scotland’s Census. (2021). Scotland’s Census 2011 - National records of Scotland. Available online at https://www.scotlandscensus.gov.uk/census-results/at-a-glance/languages/

[ref65] Shen C. Jiang W. (2023). Parents’ planning, children’s agency and heritage language education: re-storying the language experiences of three Chinese immigrant families in Australia. Front. Psychol. 13:1083813. doi: 10.3389/fpsyg.2022.1083813, 36710788 PMC9881589

[ref9007] Shih S. M. (2011). The concept of the Sinophone. Modern Lang. Assoc. 126, 709–718.

[ref66] Spolsky B. (2004). Language policy. Cambridge: Cambridge university press.

[ref9008] Stats N. Z. (2018). Languages spoken for New Zealand (2018 Census). Available online at: https://www.stats.govt.nz/tools/2018-census-place-summaries/newzealand#languages-spoken

[ref9009] Statistics Canada. (2022). Languages spoken at home by mother tongue, immigrant status and period of immigration and first official language spoken: Canada, provinces and territories and census metropolitan areas with parts. Available online at: https://www150.statcan.gc.ca/t1/tbl1/en/tv.action?pid=9810030101

[ref67] Spolsky B. (2012a). Family language policy–the critical domain. J. Multiling. Multicult. Dev. 33, 3–11. doi: 10.1080/01434632.2011.638072

[ref68] Spolsky B. (2012b). The Cambridge handbook of language policy. England: Cambridge University Press Cambridge.

[ref69] Tang X. Zheng Y. (2023). Unpacking complex language ideologies toward heritage language maintenance: a case of Chinese migrant families in the US. Int. Multiling. Res. J. 17, 333–350. doi: 10.1080/19313152.2023.2209358

[ref70] Tannenbaum M. Howie P. (2002). The association between language maintenance and family relations: Chinese immigrant children in Australia. J. Multiling. Multicult. Dev. 23, 408–424. doi: 10.1080/01434630208666477

[ref9010] Tricco A. C. Lillie E. Zarin W. O\u0027Brien K. K. Colquhoun H. Levac D. et al. (2018). PRISMA extension for scoping reviews (PRISMA-ScR): checklist and explanation. Ann Inter Med. 169, 467–473. doi:10.7326/M18-0850 30178033

[ref71] Tollefson James W. 1991. “Planning language, planning inequality.” Available online at: http://tesl-ej.org/wordpress/issues/volume1/ej01/ej01r2/

[ref9011] United Census Bureau. (2015). Detailed languages spoken at home and ability to speak English for the population 5 years and over: 2009-2013. Available online at: https://www.census.gov/data/tables/2013/demo/2009-2013-lang-tables.html

[ref72] Valdés G. (2000). Introduction. Spanish for native speakers. AATSP Professional Development Series Handbook for Teachers K, Spanish for native speakers, ed. Anderson, N. Belmont, California: Thomson. 1, 1–20.

[ref73] Valdés G. (2001). “Heritage language students: profiles and possibilities” in Heritage languages in America: Preserving a National Resource. eds. Peyton J. Ranard J. McGinnis S. (McHenry, IL: Center for Applied Linguistics (CAL)).

[ref74] Valdés G. (2005). Bilingualism, heritage language learners, and SLA research: opportunities lost or seized? Mod. Lang. J. 89, 410–426. doi: 10.1111/j.1540-4781.2005.00314.x

[ref75] Van Deusen-Scholl N. (2003). Toward a definition of heritage language: sociopolitical and pedagogical considerations. J. Lang. Identity Educ. 2, 211–230. doi: 10.1207/S15327701JLIE0203_4

[ref9012] Van Eck N. J. Va Waltman L. (2023). VOSviewer (Version 1.6.19) [Computer software]. Available online at: https://www.vosviewer.com/download

[ref76] Visonà M. W. Plonsky L. (2020). Arabic as a heritage language: a scoping review. Int. J. Bilingual. 24, 599–615. doi: 10.1177/1367006919849110

[ref77] Wang Z. (2021). Addressing migrants’ well-being during COVID-19: an analysis of Chinese communities’ heritage language schools in Germany. Migr. Stud. 9, 1144–1165. doi: 10.1093/migration/mnaa033

[ref78] Wang Y. (2023). Speaking Chinese or no breakfast: emotional challenges and experiences confronting Chinese immigrant families in heritage language maintenance. Int. J. Bilingual. 27, 232–250. doi: 10.1177/13670069221126043

[ref79] Wang L. Hamid M. O. (2022a). Journey towards an unreachable destiny: parental struggles in the intergenerational transmission of Chinese as a heritage language in Australia. Int. J. Sociol. Lang. 2022, 207–234. doi: 10.1515/ijsl-2021-0066

[ref80] Wang L. Obaidul Hamid M. (2022b). The role of polymedia in heritage language maintenance: a family language policy perspective. J. Multiling. Multicult. Dev. 45, 4139–4153. doi: 10.1080/01434632.2022.2142233, 41307611

[ref81] Wiley T. G. (2001). “On defining heritage languages and their speakers” in Heritage languages in America: Preserving a National Resource. eds. Peyton J. K. Ranard D. A. McGinnis S. (Center for Applied Linguistics/Delta Systems).

[ref82] Wu Y. (2012). Modern Chinese National-Cultural Identity in the context of globalization. J. Global Cultural Stud. Transtext (e) s Transcultures 跨文本跨文化. 7. doi: 10.4000/transtexts.456

[ref83] Wu M.-H. Leung G. (2022). ‘It’s not my Chinese’: a teacher and her students disrupting and dismantling conventional notions of ‘Chinese’ through translanguaging in a heritage language classroom. Int. J. Biling. Educ. Biling. 25, 1811–1824. doi: 10.1080/13670050.2020.1804524

[ref84] Yiakoumetti A. (2022). Teachers’ language use in United Kingdom Chinese community schools: implications for heritage-language education. Front. Psychol. 13:899428. doi: 10.3389/fpsyg.2022.899428, 36033046 PMC9403898

[ref85] Yu B. Hsia S. (2019). Inclusion of heritage language learners on the autism Spectrum: lessons from second-generation parents. Int. J. Appl. Linguist. 29, 356–369. doi: 10.1111/ijal.12233

[ref86] Zhang J. (2009). Mandarin maintenance among immigrant children from the people’s republic of China: an examination of individual networks of linguistic contact. Lang. Cult. Curr. 22, 195–213. doi: 10.1080/07908310903308279

[ref87] Zhang D. (2010). Language maintenance and language shift among Chinese immigrant parents and their second-generation children in the U.S. Bilingual Res. J. 33, 42–60. doi: 10.1080/15235881003733258

[ref88] Zhang D. Slaughter-Defoe D. T. (2009). Language attitudes and heritage language maintenance among Chinese immigrant families in the USA. Lang. Cult. Curr. 22, 77–93. doi: 10.1080/07908310902935940

[ref89] Zhou Y. Liu Y. (2023). Theorising the dynamics of heritage language identity development: a narrative inquiry of the life histories of three Chinese heritage speakers. Lang. Educ. 37, 383–400. doi: 10.1080/09500782.2022.2068351

