# Supplementary material for: Heritage language maintenance in the Inner Circle: a scoping review of Chinese and its varieties
Source: Front Psychol. 2026 Feb 23;16:1719014. doi: 10.3389/fpsyg.2025.1719014 (PMC12968234; doi:10.3389/fpsyg.2025.1719014)
Supplement: Supplementary file 1 [file Table_1.pdf]

Appendix A

**Terminology**	**Reference**	**Definition**
Heritage language speakers	Valdés (2000)	Highlighted three crucial characteristics of heritage language speakers as following:<br>1) Someone who has been raised in a home where a language other than the dominant language of the broader community is spoken,<br>2) Someone "who speaks or merely understands the heritage language",<br>3) Someone who is to some extent bilingual in the dominant language and heritage language, whether simultaneously or sequentially acquired both languages.
Heritage language learners	Van Deusen-Schooll's (2003, p. 222)	Who "have been raised with a strong cultural connection and heritage motivation to a particular language through family interactions".
Heritage language students	Valdés (2001, pp.37-38)	Highlighted two types of heritage language students:<br>1) Individuals have a historical or a personal connection to<br>a language that is not taught in schools, such as an endangered indigenous language or an immigrant language.<br>2) Individuals learn the language in a foreign language<br>classroom, individuals who are raised in homes speaking a<br>language other than English, individuals are to some extent<br>bilingual in English and the HL, and they can speak or<br>merely understand the heritage language.
