# Supplementary material for: Heritage language maintenance in the Inner Circle: a scoping review of Chinese and its varieties
Source: Front Psychol. 2026 Feb 23;16:1719014. doi: 10.3389/fpsyg.2025.1719014 (PMC12968234; doi:10.3389/fpsyg.2025.1719014)
Supplement: Supplementary file 2 [file Table_2.pdf]

Appendix B

Country	Publication Year	Authors	Participants	Research context	Focused Chinese variety	Generated Findings
Ireland	2020	Diskin, C.	45 migrants in total (23 Polish and 22 Chinese), 39 were 1st generation, 4 were 2nd generation, 2 were 1.5 generation length of residence ranging from 1-20 yrs. Participants ranges in age from 19-49, majority in early 20s and 30s, (mean age=29.7) All speak Polish or Chinese as their L1, or one of L1s.	Naturalistic	Chinese (mentioned Cantonese and Hakka, in favour of Mandarin)	1. a content analysis reveals that heritage language maintenance is of paramount importance for first, second and "Generation 1.5" migrants. 2. Weekend schools are hard to access, immersion classrooms run by Dublin Confucius Cass for children is available for enrolment for school aged children. 3. Interview illustrates that resourcefulness of migrants to maintain Chinese, Mandarin, however their respective heritage variety of Chinese is Cantonese and Hakka. 4. relies heavily on digital media sources for practical information.
Australia	2002	Tannenbaum, Michal; Howie, Pauline	40, 9–12 year-old children from Chinese-speaking immigrant families in Sydney, Australia (restricted sample to families who had been 15 years or less in Australia in order to ensure that a language ‘ dilemma'is still a living issue for most parents.)	Mixed (Home and naturalistic)	Chinese (Not specified)	1. children's perceptions of their family relations are associated with, and may predict to some extent, their tendency to use and prefer to use their parents' language. 2. higher Attachment scores (at least on the ‘ self'measure) to be associated with higher rates of language maintenance, as predicted. higher Self-reliance scores tend to be associated with lower preference for the parents' language3. It seems that combining ‘ self'and ‘ other' into one predictor score may have blurred the relationship between attachment and language maintenance. The raw correlations between the dependent variables and the ‘ self'scores for both Attachment and Self-reliance scales suggest the existence of a relationship between attachment and language maintenance that was not reflected in the multiple regression analyses.

Australia	2009	Zhang, Jingning	6 children (10 to 17 yrs old) from these 17 families (in a pilot study (Zhang, in press), interviewed 12 parents) (a group of second- and 1.5-generation (US born or arriving before age 13, respectively) immigrant children from the People's Republic of China (PRC)).	Mixed	Chinese (mentioned and noticed other varieties during interviews, but not focused on)	1. HL maintenance is not solely the responsibility of Chinese immigrant families and communities. 2. *Public school teachers and children's peers* can both be positive and negative forces towards children's bilingualism (Souto-Manning, 2006. 3.*Peer groups,* while presenting themselves as an overwhelming pull towards English assimilation, can nonetheless have a positive impact on children's HL maintenance that might be overlooked by parents and teachers. 4. *Synergies between public and community-language schools* might be a key to HL maintenance. 5. *Ethnic media has the greatest unexplored potential.*
Australia	2012	Morgan, L.; Chodkiewicz, A.	15 Chinese speaking (Mandarin, Cantonese and Chinese Vietnamese bilingual), mothers and carers with pre-school aged children, living in in an inner-city area of Sydney. (Of these 8 said they spoke Mandarin, 5 Cantonese and 4 Vietnamese and Cantonese.)	Naturalistic (play groups)	Yes (Cantonese and Chinese Vietnamese)	First, all mothers emphasized home language maintenance importance while desiring English proficiency for their children, with Area 2 mothers showing particular concern about language loss post-school enrolment. Second, participants prioritized home use of Chinese or Vietnamese through multiple strategies, including rhymes, poems, and intentional creation of language-rich environments using audio-visual materials. Third, socio-economic disparities influenced satisfaction levels, with better-resourced Area 1 mothers more critical of programs than Area 2 mothers who faced greater resource limitations. Fourth, bilingual playgroups successfully provided safe spaces for home language practice and parental guidance on bilingual upbringing.

Australia	2015	Mu, Guanglun Michael; Dooley, Karen	230 young Chinese Australians (age ranged between 18 and 35, with a mean age of 25; 111 participants were born outside Australia, with 95 born in China (the Chinese Mainland, Hong Kong, Macau and Taiwan) and 16 born in other countries (Indonesia, Malaysia, New Zealand, Singapore and Vietnam).) The Australian-born group consisted of 119 participants, with 73 identifying as second-generation, 31 as third-generation and the remaining 15 as fourth- or further removed generation.	Mixed	Chinese (Not specified)	1. Our preliminary analysis of selected demographic variables (place of birth, age of immigration and generation) revealed that each of perceived family support for CHLL, family language policy and family-supported formal CHLL, individually, was in significant relation to self-reported CHL proficiency. 2. Reproduction of habitus is not established in a vacuum but is intergenerationally sponsored, emphasising the importance of the early familial environment for CHLL. 3. A dynamic of encouragement seems to be at play also in a second form of familial inculcation evident in our data – informal CHL instruction. 4. Resistance is to the fore in participants' talk about formal Chinese learning in their school years. Interestingly, participants reportedly became committed to CHLL though it was once an unpleasant activity imposed on them. 5. participants' habitus was explicitly linked to past upbringing, present moments and future anticipations that come to shape their Chinese identity.
Australia	2020	Liao, Wanyu; Huang, Hui	3 cross-cultural families in Australia whose children attend Chinese community schools (i.e. grade 4/5), and are learning Chinese as one parent's HL (ages range from 30-50yrs old).	Mixed (home and language school?)	Chinese (Not specified)	The results suggest that, in English countries, Chinese-background and non-Chinese-background parents in cross- cultural families have quite different opinions about their children learning Chinese, which are reflected in their dissimilar language management strategies. The results highlight the importance and challenges of developing a stable family language policy in cross-cultural families in order to maintain their children's HL learning.

Australia	2021	Shen, C.; Jiang, W.	30 10-11 years old children of Chinese immigrants in Australia (consisting of 19 boys and 11 girls, aged either 10 or 11) They were born in Australia (two in New Zealand) and were attending Australian primary schools while their parents all originally came from mainland China.	Mixed (home and language school)	Chinese (Not specified)	High-achieving participants displayed positive attitudes toward Chinese learning and appreciated their study at weekend Chinese school. In regard to the expectations of their Chinese levels, the high-achieving participants expressed different degrees of aspiration toward achieving Chinese literacy. All the high-achieving participants expressed satisfaction with learning Chinese at weekend Chinese school. all the high-achieving participants reflected that they had received substantial HL support. In accordance with their reports, both parents spoke mostly Mandarin at home and provided different resources for Chinese literacy, such as Chinese television programs, books, and dictionaries. An overall reluctance to learn and use the Chinese language was prevalent among the low-achieving participants, who did not display any positive attitude toward Chinese learning and provided a variety of reasons for disliking weekend Chinese school.
Australia	2022	Wang, L.; Hamid, M.O.	quantitative survey involving 100 parents, 15 participants (14 females, one male) for follow-up interviews.	Mixed (Home, naturalistic)	Chinese (Not specified, mainly Mandarin)	Positive CHL attitudes coexisted with varied transmission commitment; parents valued CHL for career and family connections within Australia's multicultural context; negative Chinese media representation created dual-identity anxieties; conflicting high-proficiency expectations met children's resistance, with escalating pressure diminishing children's CHL value awareness; parent-child identity divergence (Chinese vs. Australian identification) intensified cultural resistance; incomplete CHL development correlated with increased cultural rejection; and parents rationalized reduced intervention as avoiding conflict, attributing lack of progress to "laziness" rather than implementing stricter approaches.

Australia	2022	Wang, Lanting; Hamid, M. Obaidul	Fifteen interviewees (fourteen females, one male) were recruited on a voluntary basis through the survey. Each of them had at least one child enrolled in mainstream education.	Home	Chinese	Parents held paradoxical views of polymedia as simultaneously beneficial (increasing HL input, maintaining family intimacy) and problematic (concerns about excessive use, limited optimization capacity); polymedia functioned as both identity carrier and barrier; parents employed mediation strategies and transcendent parenting to facilitate CHL engagement through family video calls and diverse digital literacy approaches addressing language exposure asymmetry; children resisted parental polymedia arrangements while mainstream media reinforced Australian identity over Chinese identity; and despite creating intergenerational tensions, parents strategically used polymedia to mediate family language policy, balancing competing interests while acknowledging child agency's impact on parenting effectiveness.
Australia	2023	Huang, Hui; Liao, Wanyu	four Chinese–English interlingual families ((1) all participants participate voluntarily (including both parents and the child); (2) these families are interlingual (i.e. one parent speaking Chinese as L1 and the other parent (only) speaking English); (3) each family has a child aged 10–12 years who was studying Chinese at the time of data collection.)	Naturalistic	Chinese (Not specified)	1) Each parent from the families held consistent views on maintaining Chinese, such as for the sake of maintaining heritage, as an instrumental function for a future career, or for the cognitive benefits of learning a new language. However, their ideas on whether, and to what extent, Chinese should be maintained were dissimilar, sometimes even contradictory. (2), all parents reported that there was no other community support, except for the Chinese community school. Home exposure and language use in the interlingual families were quite limited (Language use journal: ranging from half an hour to three hours a week). However, each participating child had an extra 3–4 h exposure to the language through attending a weekly 3-h class at the weekend, and completing assigned homework in 1–2 h. Chinese community schools provided support for children's Chinese learning; the degree to which community school contributed to these children's language learning was largely determined by how the family perceived the school and supported school tasks in action. (3), The language use, the level of self-efficacy, and child agency were found with connections to children's confidence in maintaining HL.

Australia	2023	Shen, Chunxuan; Jiang, Wenying	The three families involved in this article were epitomes of the 30 families the researchers recruited for a larger project.	Weekend Chinses school	Cantonese, Shanghainese	1. Parent agency in FLP varied from each other, disparities between the three stories have evidenced the remarkable contribution of family support to the child's HL competence. The quality of HL language input and the influence of HL literacy experiences demonstrate to be crucial (Sun, 2019). The early HL exposure, ongoing commitment to HL use and literacy-based HL activities initiated by parents contribute to children's confidence and competence in HL. 2. Children's language ideologies are shaped and negotiated in their everyday language practices at home with their parents. Immigrant parents tend to have the intention to transmit their HL and use explicit language practice and management strategies to influence their children's language development (Park and Sarkar, 2007; Szecsi and Szilagyi, 2012, etc.). However, children may contest or resist their parents' efforts and undermine their parents' FLP (Mu and Dooley, 2015; Smith-Christmas, 2022). 3. parents valued Mandarin over dialects such as Cantonese and Shanghainese, Leo is the only child who had a harmonious relationship with his family when it comes to FLP.

Australia	2023	Wang, Yining	12 Chinese immigrant families including 13 parents and 13 children. The study focuses on parents who brought their children into Australia when they were between ages 3 and 9.	Naturalistic	Chinese (Not specified)	Findings show that maintaining heritage language in immigrant and minority contexts is fraught with negative emotions such as irritation, regret, guilt, anguish, loss, insecurity, and shame, but this is complemented by a sense of accomplishment, fulfilment, and pride. It is language anxiety or negative emotions related to heritage language maintenance that predominates in parental discourse, due to the gap between their expectation/ children's previous Chinese literacy competence in China and parents' lack of success in their maintenance endeavours as well as their children's language attrition. Parental emotions, whether positive or negative, which are shaped by language ideologies of Chinese as identity, family tie, and profit, are often elicited by children's language behaviours and their proficiency outcomes. Such values, moral standards and life styles could be instilled by carefully selecting literature for children to read. The study suggests that children's heritage language proficiencies are significant for the wellbeing of immigrant families.

New Zealand	2020	Li, Sophia	Anna is a 38 year-old housewife, with her two sons, 8 years old King and 3 years and 7 months old Jimmy. King goes to a primary school in West Auckland and Jimmy goes to a kindergarten in the same area.	Home	Chinese (Not specified)	1. Anna explained her understanding of multilingualism that it is beyond ethnicity and boundaries, “It is not about where you are from or what language you speak. It’s just simply the fact that we speak two languages, so why don’t we support our children to develop in two languages?!” “It’s not about the concept of ethnicity or the boundary between countries.” (Anna: Interview 2, p. 3)" 2. Anna shared power and authority at home with her children, and she did not seem to feel embarrassed or awkward when being helped by the two young children. When their FLP is focused on supporting the children’s Chinese language development, the children are keen to help Anna with her limited English proficiency at the same time. 3. Anna explained that Mandarin Chinese is the only language spoken at home among all family members. She justified speaking Mandarin for communicative purposes to the children, she is being very cautious with these two boys’ individual learning traits and needs. She is aware of the differences between the learning styles of her two young children, and she is trying to adopt appropriate language practices to support their dual language development.

United Kingdom	2018	Curdt-Christiansen, Xiao Lan; La Morgia, Francesca	28 Chinese families with at least one child aged between 2 and 8 years (N = 66). 13 girls, 15 boys; 10 children were under 3yrs, 10 children aged 3-5 yrs, 8 children were over 5yrs.	Home	Chinese (Not specified)	1. The linguistic practices in the family domain can illuminate the process of language change and the patterns of language practices determined by family members' conscious or unconscious choices. 2. English is not the primary medium of interaction for the Chinese parents, as 86.2％(N = 25) of them reported using less than 25％of English in their linguistic repertoire. 3. Children in the Chinese families also tend to use less English when interacting with their siblings, as studies of intergenerational transmission have reported otherwise (Curdt-Christiansen 2016; He 2013; He 2016). This may be explained by the children's delayed exposures to English and their young age, as nine out of the 28 children were cared for at home. 4. parents permitted varied hours of English TV programs for children, while only 4 families provided HL TV programs for their children, similar to educational games. 5. Variation in frequency of reading in English and HL was found, similar to the frequency of visiting a library and the distribution of English and HL books at home. 6. Chinese parents and Urdu-speaking Pakistani parents seemed to have higher expectations of explicit reading and writing skills for their children whereas the Italian parents tended to give more emphasis on communicative abilities. The Chinese parents saw more importance in formal literacy skills such as "comprehension ability", "can speak in full sentences" and "calculation skills".

United Kingdom	2019	Wei, Li; Hua, Zhu	a further subset within the 30 three-generational families; for interviews after 10 ten years: two maintenance families : M1, M4; five shift familes: S6, S9, S10, S12, S13 (18 individuals)	Naturalistic	Cantonese, Hakka	1.Five families (M1-M5, three originally Cantonese-speaking, two Mandarin-speaking families = 37 individuals) that seemed to have maintained their original languages, measured in terms of using it as the primary language of inter-generational interaction, and eight families (S6-S13, five originally Cantonese including two with some Hakka, one Hakka, and two Mandarin = 61 individuals) that have undergone major language shifts were selected and invited to talk about the two topics in interview-style conversations with the researchers. 2. For the families that have by and large maintained the heritage language for their intergenerational interaction, three themes came out from the participants' responses to our interviews: – the grandparent factor; – desire to return to place of origin; – future prospect and opportunities for the children; – dissatisfaction with life in the UK. 3. For the families who experienced significant shift in habitual language use towards English, several common themes were articulated, often together in one account. The themes include: – easier, but not necessarily happier, life in the UK; – friendship ties in the UK; – strong desire for the children / younger generations to live a better life and speaking English can help them to gain access to better life; 4.the vision of the future of the children of the family is closely intertwined with their migration experience, their present position in the place of residence, and their relationships with other families and social groups. 5. the role of broader family relations in the participants' imaginations. 6. families who shifted from Chinese to English gave reasons of past experience or expectations for future employment, as some of the examples in the sections above show. Others expressed their feeling of fatalistic inevitability. 7. chances and opportunities 8. new diasporic thinking the global connections, with relatives in different parts of the world, enhance their desire for more mobility and connectivity, rather than stability in the traditional sense.

United Kingdom	2022	Yiakoumetti, Androula	four teachers (In compliance with the preferences of the heads of each school, teachers who taught Beginners' Mandarin Chinese were chosen.) The teachers originated from mainland China with Mandarin being their first language and English a foreign language.	Community school	Chinese (Not specified)	1. Unexpectedly, analysis of teachers' language use revealed that they primarily used students' dominant L1 (English) during lessons; All teachers systematically reverted to students' dominant language, English, to introduce and explain new vocabulary and grammatical structures.2. All teachers reported that they systematically and consciously harnessed students' L1. Their individual comments had a common theme: awareness that the L1was regularly employed for the smooth running of every lesson. teachers struggled to determine how much English vs. Mandarin to use during lessons, Teachers expressed a desire for training in issues relevant to ethnic-language education. 3. the role of family to be at the forefront of successfully maintaining and developing heritage-language competence, technology may play a key role in the learning of ethnic languages for diasporic communities, This study provides evidence that teachers should ideally undergo training in how to teach ethnic minority languages.
United States	2003	Yan, Ruth Lingxin	65 CLD parents (16 Chinese parents) who chose to send their children to language schools.	Mixed (Home and language school)	Chinese (Not specified)	(1) The oral heritage language was used by the majority of CLD students' parents at home except by the parents from the Hebrew language group, (b) the CLD students' held positive attitudes toward heritage language learning, and (c) the CLD students' parents believed that ideal quality schools for their children were bilingual schools or those that provided instruction with extra heritage language teaching. The interview data showed that the main reasons for CLD students' parents to maintain their children's heritage language learning were as follows: (1) to maintain cultural and religious heritage; (2) to strengthen family ties and moral values; (3) to keep connections to their own cultural and language communities; and (4) to promote bilingual skills for better job opportunities.

United States	2009	Zhang, Donghui; Slaughter-Defoe, Diana T.	16 first-generation parents The children include four boys and 14 girls, and their ages range from six to 14, i.e., from first graders to eighth graders. Six children are from Fujianese-speaking families and 12 from Mandarin families.	Mixed (home, language school, naturalistic)	Fujianese	(1) Chinese parents generally held positive attitudes towards HL, for a) home language as an important resource that their children could take advantage of in their academic advancement and future career. b) home language as closely related to their ethnic identity, an important heritage that connects the second-generation children to their home country and culture. c) as a necessary family link that reinforces family ties and contributes to family cohesion. Chinese parents seek to transmit ethnic identity to their children, they look to the HL to do it, they recognize Mandrin as the most significant characteristic of their ethnic group. (2) Using Chinese as the home language; Becoming heritage language teachers; Cultural participation and Chinese weekend schools (3) Most Chinese children, especially older children, feel Chinese is something they have to learn to obey their parents, yet they themselves do not feel it necessary or important (However, the enthusiasm follows a descending pattern with age.); Children's perception of the HL being 'useless' reveals a lack of support for HL learning in the mainstream American schools and in the larger society.

United States	2010	Zhang, Donghui	18 Chinese immigrant families (20 parents involved since in some cases both parents were present for the interview. The majority of the parents were mothers (15, or 75%)) 4 boys and 14 girls (6-14 yrs old), Six children were from Fujianese-speaking families and 12 from Mandarinspeaking families	Mixed	Fujianese	(1) The Mandarin parents typically reported that they were more comfortable speaking Chinese than English because it gave them a sense of familiarity and closeness, and allowed them a wider space to express themselves freely. While the Fujianese immigrants relied on the heritage language (either Fujianese or Mandarin) by necessity, (Structural barriers form a vicious cycle, preventing the Fujianese immigrants from successfully acculturating in the host society and leading them to rely on their heritage language "by necessity"), it seems that the Mandarin parents used the heritage language by choice. (2) Compared with the Mandarin children whose parents were bilingual, the Fujianese children found themselves obliged to speak Fujianese at home in order to maintain connection with their parents as well as other ethnics in the neighbourhood of Chinatown. However, their heritage language maintained out of necessity was rather limited and not as satisfactory as expected. Learning English, therefore, was emphasized to the next generation as the route to achieve social mobility. Compared with the Fujianese children, the Mandarin children used their heritage language less frequently. At home they were not restricted to the heritage language as the only means of communication because their parents understood them in both English and Mandarin Chinese. Therefore, the heritage language became a language to be used on limited social occasions.

United States	2012	Zhang, Donghui	18 Chinese immigrant families (mandarin speaking and Fujianese-speaking)	Mixed	Fujianese	(1) Interviews with the Mandarin families revealed that the passive ties in the home country were still meaningful to them in the new land. They maintained regular contact with their immediate relatives, friends, and colleagues over time and exhibited transnational behaviour, such as cross boarder communication, on a routine basis. (2) Co-ethnic network as exchange ties and as interactive ties: they invariably identified co-ethnic Chinese as their significant ties in the host country, whereas associates in cross-ethnic networks were rarely seen as "friends, the co-ethnic network among the Mandarin families was of a high-density nature in which closeness, warmth, and mutual help were typically found. " the Fujianese belong to a close-knit and high-density co-ethnic network in Chinatown, typical for lower-class, poor communities that Milroy (1982) characterized. (3) Although the Mandarin parents in my study all had the intention to maintain the HL to the next generation, the second-generation children who socialized with non-Chinese peers at school and in the larger society questioned the necessity of learning Chinese in the United States. Because of huge assimilation pressures at school and children's sensitive feelings particularly during adolescence, HL learning became a major headache and caused intergenerational conflicts in many Mandarin families. In response to the negative comments from non-Chinese classmates, the Fujianese children tended to form high-density co-ethnic networks as a coping strategy for collective survival in the U.S. society; however, the HL was not welcome among the Fujianese children. Even though they lived in Chinatown and were immersed vis-à-vis the co-ethnic community every day, they did not want to inherit the language.

United States	2013	Yu, Betty	10 bilingual, Chinese/English-speaking, immigrant mothers and 5 fathers with their children with autism spectrum disorders.	Naturalistic	Chinese (Not specified)	1. In short, all of the parents prioritized the language that was dominant in the society in which they intended to reside, that language being English. All of the participants valued Chinese highly even though they were diverse in their opinions about the degree to which their children needed to learn it. 3. A clear priority for all of the parents in the study was to address the perceived life barriers that they associated with the autistic condition. If the heritage language was perceived to be an obstacle to that goal, then it was minimized or dropped. 4. The biggest challenge identified by the mothers was that very few interventions were available in Chinese. 5. The parents' views of their children's language needs were tied to their sense of self-efficacy when it came to supporting their children's development, yet most of the parents claimed that they didn't know how to support children's intervention. 6. All of the mothers in the study expressed some level of reservation about the effects of bilingualism on their children. Even the parents who spoke to their children bilingually expressed that all things being equal, they believed a monolingual environment would be better. 7. conflicting advice received from professionals, most of the professionals suggest they should start speak in English only as soon as possible, few suggested that they should speak the mother tongue (i.e. in this case, Chinese). 8. A lack of versatility of speaking in English was found in most of the interviewed responses, and the ability to communicate in English heavily depended on the context (professional or casual).

United States	2019	Yu, Betty; Hsia, Summer	The three participants in the current study – Amy, Curtis, and Nathan1 – were part of a larger pool of fourteen participants in an ongoing survey study of parents who live in the U.S (a), have children on the autism spectrum (b), and identify Chinese as a heritage language (c), they identified as secondgeneration children of Chinese immigrants.	Mixed	Chinese (Not specified, but Cantonese was mentioned in Amy's interview)	Findings showed that heritage language learning was highly valued by all three parents, but parents whose children were more severely affected by autism prioritized autism remediation over language education. We also found a lack of educational and social opportunities for addressing the intersectional needs for both heritage language learning and disability supports. Furthermore, these gaps existed both in the English-speaking and Chinese-speaking communities, which created a sense of double isolation/exclusion among the parents. Specifically, we argue for an inclusive approach to education premised on sociocultural grounded and intersectional understandings of disability, culture, and language. The shifts in the parents' practices were informed and shaped by the contextual constraints in which they and their children were embedded
United States	2020	Kaveh, Yalda M.; Sandoval, Jorge	Eight families,including six two-parent families and two single-parent families. All were first-generation immigrants. One child and one parent participated in each family (N = 16). The participants consisted of five fourth-grade children (one girl and four boys) and their parents (one father and four mothers) at the urban school (N = 10) and three children (one boy and two girls) and their parents (three mothers) at the suburban school (N = 6).	Mixed (Home and governmental school)	Mandarin	1. Our findings showed that the participating parents' and children's spoken beliefs consistently positioned bilingualism and heritage language maintenance as valuable. However, family language practices showed an increasing preference for English that was alienating heritage languages. 2. Despite the schools' verbal encouragement for home language use, a few of the participating parents still experienced internal tensions about their language choices at the advent of schooling. 3. Families in both urban and suburban contexts utilized a variety of strategies to maintain heritage language use in spite of evolving language practices, findings did not suggest a significant difference in language management based on the families' background, such as neighborhood, country of origin, length of parents' residency in the U.S. or their socioeconomic status indexed by education level, job status, and self-assessed English proficiency. 4. Based on all eight parents' descriptions, their push for heritage language use was met with 1) resistance from their children, 2) conspicuous dominance of English in their households, and/or 3) the children's increasing comfort with English.

United States	2021	Leung, G.	93 Hoisan heritage people aged 8–97 in the San Francisco Bay Area between 2010 and 2012.	Naturalistic	Cantonese, Lliyip, and Hoisan-wa	a) participants' anchorings of Chinese varieties; shift from Hoisan-wa to standard Cantonese to Mandarin indicated a new orders indexicality (Blommaert, 2005). Spatial anchoring was also evident when speakers' deep connection with particular places and forms of language, while offering attributes of the place where they are anchored to. though admitting the "sameness" between Cantonese and Hoisan-wa, speakers tended to exclude themselves from Hoisan-wa speakers by comparing the differences from geographical to linguistic features. b) self-reported fluency data; The youngest generation had the lowest reported fluency (5.136 out of 7, hovering around "can understand and speak a few sentences"). The middle generation reported an average of 2.686 out of 7, and the oldest generation reported an average of 1.583, making them the most fluent group in Hoisan-wa/Lliyip. The average reported fluencies of Hoisan-wa/Lliyip and Cantonese were similar at 2.839 and 2.936, respectively. Taking a closer look at Cantonese, the youngest generation gave themselves a higher fluency score than they did for Hoisan-wa/Lliyip: 4 out of 7 ("can understand and speak simple sentences"). As age increased, so did the average fluency in Cantonese. The middle generation reported an average of 2.714 out of 7, nearly the same as their average for Hoisan-wa. The oldest generation reported an average of 2.5 out of 7. The average reported fluency for Mandarin is 5.620 out of 7, with the oldest generation reporting the least fluency in Mandarin. The mean differences between the young (5.136 out of 7) and middle (5.200 out of 7) generations were not significant, meaning both groups' reported fluencies were about the same. c) the use of the term "Chinese. Yet this point is important to note, serving as a reminder that current "Chinese-as-Mandarin" discourses are indeed arbitrary in nature and contesting these "common sense" notions of language by giving voice to those at the "periphery".

United States	2021	Liang, Feng; Shin, Dong-Shin	three families (All of the participating families lived in the suburbs and stated themselves to belong to the middle-class. Although fathers played an important role in HLM, mothers accompanied the children to the CHL school most times and agreed to become parent participants in the study. All the mothers could speak English and Mandarin fluently as first-generation immigrants and had completed BA degrees in China before immigrating. The child participants were all born and raised in the United States,)	Naturalistic	Chinese (Not specified)	1. Three families upheld HLM for various reasons including family communication, ethnic and cultural identity, and practical considerations such as education and career, but the families shared family communication as the primary goal. 2. Only children (i.e., Kevin and Jason) expanded the purpose of HLM into communications with Chinese friends or neighbours in the community beyond family members and relatives although they preferred to speak English with Chinese-speaking friends in the CHL school and in the community. Only parent participants explicitly mentioned their Chinese ethnic identity (i.e., Amy and Sandy) and practical considerations (i.e., Penny and Amy) as a reason for HLM. 3. All three families had established implicit and explicit family language policies for HLM and utilized various language learning resources. 4. Regarding the challenges that the participants faced in HLM, all three families mentioned limited time and energy for learning Chinese due to the children's increased extracurricular activities.

United States	2022	Smith, Sara A.; Li, Zhengjie	Fifty-eight children enrolled in weekend Chinese Language school, ages 10–18 (28 females; M = 12.93 years, SD = 1.82 years).	Mixed (home, Weekend Chinese language school, digital environment)	Chinese (Not specified)	1. There was a significant positive correlation between ideal HL self and the measure of intended effort in school (r = .35, p = .008); Current Chinese-language self was significantly correlated with self-reported Chinese language ability: speaking (r = .622, p = .000), listening (r = .549, p = .000), reading (r = .367, p = .005), writing (r = .269, p = .041). However, there were no significant relationships between self-reported Chinese ability, in any modality, and intended effort in school. 2). all statements regarding English had significantly higher agreement than the Chinese language counterpart statement; There were no significant positive relationships between Chinese and English reading statements and no significant relationships between English reading attitude statements and intended Chinese language school effort, or Chinese language motivational facets; Intrinsic enjoyment of Chinese reading was positively associated with intended classroom effort, current HL self, and ideal HL self; so did extrinsic value of reading 3). Children reported more use of English in digital settings than Chinese; however, participants but did report Chinese use in digital environments.

United States	2022	Wu, Ming-Hsuan; Leung, Genevieve	Teacher Meihua (a pseudonym) (female) roughly 200 K-8 graders (about 40% of the total student population) in the heritage track (one-third of her students were from Fujianese-speaking families, one-third from Cantonese-speaking backgrounds, and the remainder from mixed backgrounds (i.e. a combination of Mandarin, Fujianese, Cantonese, Taishanese, Indonesian, or Vietnamese))	Language school	Mandarin, Cantonese, Fujianese, Taishanese, Hakka, or a combination of these languages.	1.Children who did not speak Mandarin at home faced difficulties stemmed not just from the mere difference between their HL and Mandarin, but were also related to their struggles of learning an ascribed HL they did not necessarily identify with, their unique needs as non-Mandarin heritage learners are not recognized. 2. The teacher learned counting from 1-10 in Fujianese from her students and realized how different Fujianese is compared to Cantonese, what happened above confirms prior translanguaging research on the importance of translanguaging in social space (Milu 2013) as well as advancing teacher-student rapport (Li and Luo 2017). 3. students who were rarely seen participating in the Mandarin classroom, had a vast knowledge base that their teacher could tap into. While Cantonese and Fujianese are not mutually intelligible to Mandarin, they share similarities in syntax and thus students' knowledge of Cantonese and Fujianese was still very helpful in learning Mandarin. 4 Meihua's willingness to learn students' home languages and openness to include discussion of different varieties enable her students to develop not only metalinguistic awareness of different Chinese varieties but also critical language awareness that challenged the hegemonic language policy in multilingual societies. we argue that translanguaging pedagogy should at least start with an acknowledgement of linguistic diversity in the Chinese diasporic communities so that a translanguaging stance can be enacted by creating spaces for multiple varieties of Chinese in the Mandarin classroom. 5. the HL classroom is full of tensions and potential mismatches in students' linguistic realities and aspirations

United States	2023	Tang, Xiruo; Zheng, Yongyan	six Chinese migrant families in Boston, U.S.	Home	Chinese (Not specified)	The findings revealed these migrant families' capability of perceiving their heritage language as a problem, a right, and a resource to cope with real-life problems they encounter in the host country. Moreover, the possible coexistence and contradiction of multiple ideological orientations within each family may be associated with the intergenerational linguistic transmission.
